# mtDNA-cGAS-STING axis-dependent NLRP3 inflammasome activation contributes to postoperative cognitive dysfunction induced by sevoflurane in mice

**DOI:** 10.7150/ijbs.91543

**Published:** 2024-03-03

**Authors:** Nan-Shi-Yu Yang, Wen-Jing Zhong, Han-Xi Sha, Chen-Yu Zhang, Ling Jin, Jia-Xi Duan, Jian-Bing Xiong, Zhi-Jian You, Yong Zhou, Cha-Xiang Guan

**Affiliations:** 1 Department of Physiology, School of Basic Medical Science, Central South University, Changsha, Hunan 410078, China.; 2 Key Laboratory of General University of Hunan Province, Basic and Clinic Research in Major Respiratory Disease, Changsha, Hunan 410078, China.; 3 National Experimental Teaching Demonstration Center for Medical Function, Changsha, Hunan 410013, China.; 4 Department of Anesthesiology, Liuzhou People's Hospital, Liuzhou 545000, China.

**Keywords:** postoperative cognitive dysfunction, neuroinflammation, cGAS-STING, mitochondrial fission, NLRP3 inflammasome.

## Abstract

The activation of NLRP3 inflammasome in microglia is critical for neuroinflammation during postoperative cognitive dysfunction (POCD) induced by sevoflurane. However, the molecular mechanism by which sevoflurane activates the NLRP3 inflammasome in microglia remains unclear. The cGAS-STING pathway is an evolutionarily conserved inflammatory defense mechanism. The role of the cGAS-STING pathway in sevoflurane-induced NLRP3 inflammasome-dependent neuroinflammation and the underlying mechanisms require further investigation. We found that prolonged anesthesia with sevoflurane induced cognitive dysfunction and triggered the neuroinflammation characterized by the activation of NLRP3 inflammasome* in vivo*. Interestingly, the cGAS-STING pathway was activated in the hippocampus of mice receiving sevoflurane. While the blockade of cGAS with RU.521 attenuated cognitive dysfunction and NLRP3 inflammasome activation in mice. *In vitro*, we found that sevoflurane treatment significantly activated the cGAS-STING pathway in microglia, while RU.521 pre-treatment robustly inhibited sevoflurane-induced NLRP3 inflammasome activation. Mechanistically, sevoflurane-induced mitochondrial fission in microglia and released mitochondrial DNA (mtDNA) into the cytoplasm, which could be abolished with Mdivi-1. Blocking the mtDNA release *via* the mPTP-VDAC channel inhibitor attenuated sevoflurane-induced mtDNA cytosolic escape and reduced cGAS-STING pathway activation in microglia, finally inhibiting the NLRP3 inflammasome activation. Therefore, regulating neuroinflammation by targeting the cGAS-STING pathway may provide a novel therapeutic target for POCD.

## Introduction

Perioperative neurocognitive disorders (PND), including postoperative delirium (POD) and postoperative cognitive dysfunction (POCD), are serious perioperative complications at an alarming rate in elderly surgical patients 1. POCD is a neurological complication that results in cognitive impairment after surgical anaesthesia, manifesting as insanity, impairment of personality, learning and cognitive abilities 2. The morbid state not only leads to a reduced quality of life, but also facilitates the exacerbation of the disease. 3. Despite enormous research efforts in recent decades, the pathogenesis of POCD is not fully understood, which is an obstacle to drug development and POCD prevention.

Neuroinflammation, principally driven by microglia, is a predominant neuropathological hallmark of POCD 4. Microglia are the resident macrophages in the brain that respond rapidly to brain injury and undergo distinct morphological changes 5. Previous studies have confirmed that microglia are critically involved in the initiation and development of POCD 6, 7. The NOD, LRR, and pyrin domain-containing 3 (NLRP3) inflammasome, which plays a fundamental role in neuroinflammation, is a cytosolic signaling complex comprising a sensor molecule, the adaptor apoptosis-associated speck-like protein containing a CARD (ASC) and the effector pro-caspase1 8. Once activated, the NLRP3 inflammasome induces self-cleavage and activation of pro-caspase1. This mediates the maturation and secretion of the interleukin-1 family, such as interleukin-1 beta (IL-1β) and interleukin-18 (IL-18) 9, 10. Our recent studies found that inhibition of NLRP3 inflammasome reduces lipopolysaccharide-induced inflammation in acute lung injury 11-13. Sevoflurane is an inhalational anaesthetic commonly used in surgery 14. Studies have shown that sevoflurane affects cancer progression and inflammation by mediating gene expression and signaling pathways 15, 16. Another investigator has identified that inhibiting the NLRP3 inflammasome in the hippocampus alleviates sevoflurane-induced POCD in aged mice 17. However, the mechanism by which sevoflurane induces the NLPR3 inflammasome activation of microglia in POCD remains unclear.

The cGAS-STING signaling pathway, comprising the cyclic GMP-AMP synthase (cGAS) and the cyclic GMP-AMP receptor stimulator of interferon genes (STING), is an evolutionarily conserved defense mechanism 18. Upon binding to double-stranded DNA (dsDNA), cGAS produces a secondary messenger called cyclic GMP-AMP (2'3'-cGAMP) to activate its downstream target STING. STING recruits TANK-binding kinase 1 (TBK1) to bind interferon regulatory factor 3 (IRF3) and then generates IRF3-dependent type 1 IFNs or promotes IRF3 and nuclear factor-κB (NF-κB) binding to release pro-inflammatory cytokines 18. In recent years, the interplay between the cGAS-STING signaling pathway and NLRP3 inflammasome has been revealed. In ischemic stroke, inhibition of cGAS can reduce NLRP3 inflammasome-induced pyroptosis of microglia 19. Research has shown that excessive STING activation causes lysosomal rupture, which can activate the NLRP3 inflammasome by releasing lysosomal K^+^ 20. Nonetheless, whether sevoflurane induces the cGAS-STING pathway-dependent NLRP3 inflammasome activation during POCD requires further investigation.

In response to cellular stress, mitochondrial DNA (mtDNA) is released into the cytoplasm, where mtDNA activates cGAS-STING to trigger inflammatory responses 21, 22. The mechanism that triggers the release of mtDNA is under investigation. Mitochondria are in a highly dynamic meshwork structure, constantly in the process of fission and fusion, known as mitochondrial dynamics 23. Mitochondrial fission has two spatially distinct types of division: one is central to the organelle, while the other is peripheral and occurs at the ends of the mitochondria 23. When mitochondria are damaged by stimulation, peripheral fission occurs, which can divide into two mitochondria of different sizes. The smaller product of peripheral fission often has defective mtDNA 23. mtDNA can be released from mitochondria into the cytoplasm *via* mitochondrial permeability transition pore (mPTP) and voltage-dependent anion channels (VDAC), activating the cGAS-STING signaling pathway 24. Mitochondrial fission depends mainly on the GTPase dynamin-related protein 1 (DRP1), a member of the dynamin family of GTPases 25. Phosphorylation of DRP1 at Ser616 encourages its recruitment to the outer mitochondrial membrane to drive fission 26. DRP1-mediated mitochondrial fission can cause structural disruption and over-opening of mPTP, exacerbating mitochondrial and cellular dysfunction following hypoxia 27. mtDNA could be released through the channel formed by the oligomerization of VDAC during DRP1-dependent mitochondrial fission, triggering STING signaling activation in LPS-treated Kupffer cells 28. Thus, mitochondrial fission would be a key mechanism inducing the cGAS-STING pathway-dependent NLRP3 inflammasome activation.

In this study, we first observed that blockade of cGAS-STING pathway activation could reduce cognitive dysfunction and NLRP3-associated neuroinflammation in sevoflurane-induced POCD mice. Mechanically, we found that sevoflurane promoted DRP1-dependent mitochondrial fission to release mtDNA into the cytoplasm *via* the mPTP-VDAC channel, which induced the cGAS-STING pathway-dependent NLRP3 inflammasome activation, resulting in neuroinflammation of microglia. Our study suggests that targeting the cGAS-STING pathway may provide a new therapeutic pathway for POCD and other neurological diseases.

## Materials and Methods

### Animal experiments

The 6-8 weeks male C57BL/6J mice were provided by Hunan SJA Laboratory Animal Co., Ltd (Hunan, China). All animal protocols were approved by the Ethics Committee of the Institutional Animal Care and Use Committee of Central South University (CSU-2023-0183).

### The POCD mouse model

**Figure 1:**
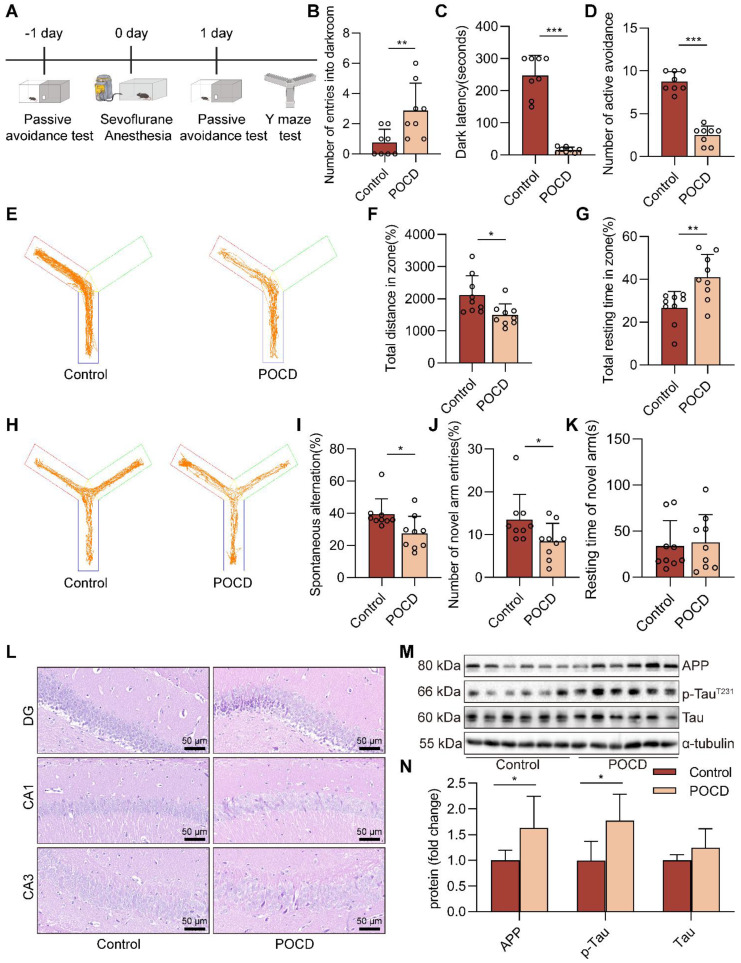
** Prolonged anesthesia with sevoflurane triggers cognitive dysfunction and neurological impairs in mice. A** C57BL/6J mice were trained in the passive avoidance test. The next day, the mice were exposed to 4% sevoflurane for 6 h to induce cognitive impairment. On the first day after sevoflurane exposure, the mice underwent the passive avoidance test and Y-maze test. **B-C** The dark avoidance test was conducted to assess the number of entering the darkroom and the dark latency (*n*=8). **D** The shuttle experiment was conducted to assess active avoidance (*n*=8). **E** Representative trajectories of each group in the training session of the Y-maze test. **F-G** Total distances and total resting times were assessed in the Y-maze training (*n*=9). **H** Representative trajectories of each group in the Y-maze testing. **I-K** The percentage of spontaneous alternation, the number of entries into the novel arm, and the duration spent in the novel arm were assessed in the Y-maze testing (*n*=9). **L** HE staining of hippocampal tissue (bar=50 µm). **M-N** Protein levels of APP and p-Tau^T231^ in the hippocampus were detected by western blot (*n*=6). Data are expressed as the mean ± SD. Comparisons between the two groups were made with an unpaired* t*-test. *P < 0.05, **P < 0.01, and ***P < 0.001.

We used 4% sevoflurane in 100% oxygen for C57BL/6J mice anesthesia, which has been reported to effectively trigger cognitive impairment in previous studies 29, 30. Mice were randomly divided into the Control, POCD, and POCD+RU.521 groups (RU.521: a specific inhibitor of cGAS). Mice in the POCD group were exposed to 4% sevoflurane (License No. H20150020, Maruishi Pharmaceutical Co., Ltd., Japan) in 100% oxygen for 6 h in an anesthetizing box by an ABS rodent anesthesia machine (Yuyan Corporation, Shanghai, China). During sevoflurane exposure, mice were placed on a heating pad support to keep body temperature within 36.5 ± 0.5 °C and observed respiration to prevent respiratory depression. After sevoflurane exposure, mice were immediately transferred to a resuscitation chamber filled with fresh oxygen. To assess the role of the cGAS-STING pathway in sevoflurane-induced POCD mice, mice in the POCD+RU.521 group were treated with RU.521 (5 mg/kg) by intraperitoneal injection 2 h before the sevoflurane anesthesia. The schematic diagram of the animals' experimental procedure is summarized in Figure 1A and Figure 4A.

### Passive avoidance test

Behavioral assessments (8-9 mice per group) were performed, including the Passive avoidance test, Y-maze test, Open field test, and Elevated plus maze. All behavioral assessments were conducted in a noiseless room. For the passive avoidance test, the mice were placed in a shuttle box and stimulated by light (or sound) and electric shock to record the active avoidance response index during the establishment of this conditioned reflex, which can reflect the changes in learning and memory ability of experimental animals 31, 32. The passive avoidance test consists of a dark avoidance test and a shuttle experiment, with an acquisition trial and a retention test 24 h later. In the dark avoidance test, before starting the training phase, each mouse was first placed in the device (the guillotine door was open) and was allowed to explore freely in both compartments for 3 min. Then, it was returned to the home cage. In the acquisition trial, each mouse was followed by turning on the current for 300 s. When it entered the dark chamber, a 0.5 mA electric foot shock (5 s) was delivered. After 24 h, the retention trial was repeated as the acquisition trial without an adaptation period. The mice were placed into the light chamber without adapting, and the dark latency (the first time the mice entered the dark chamber) and the number of times they entered the dark chamber was recorded during the 300 s test period. During the training phase of the shuttle experiment, the mice were placed in the shuttle box, and the experimental parameters were set as light flashing for stimulation, 0.5 mA electric shock stimulation for 5 s, a total duration of 15 s, and 15 shuttle cycles. Repeat the operation of the acquisition experiment after 24 h as the result of the retention experiment. If the mice escape to the safe area during the light flashing stimulation, they are regarded as active avoidance. If a mouse escapes to a safe area during electrical stimulation, it is considered passive avoidance. The number of active evasions to assess learning and memory ability was calculated as the number of shuttle cycles minus the number of passive evasions.

### Y-maze test

The Y-maze test is widely used to study spatial learning and memory 33, 34. The test is conducted using a Y-shaped maze with three wooden arms, which was performed by marking each arm as a starter arm, a familiar arm, and a novel arm. The recognition abilities of the mice were measured by blocking off one arm of the Y-maze (the novel arm), and their spatial working memory was assessed by recording the spontaneous alternations. In this study, the test consists of training and test sessions. For the training session, the novel arm was closed, and the mice were placed in the starter arm, allowing free access to the familiar arm for 5 min. After 2 h, during the test phase, the animal was again placed on the starter arm, and its preference for the novel arm or known arms was observed for 5 min. The Smart3.0 was used to record how long the mice stayed in the novelty arm, the number of times they entered it, and the number of times they entered the unrepeated consecutive arm (i.e., ACB, BAC, CAB).

### Open field test

The Open field test can be used to assess spontaneous movement in mice 35, 36. Mice are allowed to freely explore a square exercise box (100 cm long, 100 cm wide, 40 cm high) for 5 min. The time spent by the mice in the central area of the square was observed and recorded. At the end of the experiment, the mice were removed, and the entire arena box was cleaned with 75% alcohol. After the box was dried, the experiment was repeated with different mice.

### Elevated plus maze

The Elevated plus maze consists of a pair of open arms (50 × 10 cm) and two closed arms (50 × 10 × 40 cm) connected by a central square (10 × 10 cm) 50 cm above the ground. Mice were allowed to wander freely in the maze for 5 minutes, while the number of times the mice entered the open arm and the duration of their stay was recorded. The time spent in the open arm of the elevated maze was measured to assess the exploration and activity level of animals, and the number of entries into the open arm was used to assess the exploratory and cognitive abilities of animals in the elevated maze.

### Hematoxylin and eosin (H&E)

Twenty-four hours after sevoflurane exposure, the brains of the mice were fixed with a 4% neutral buffered formaldehyde solution. Brain slices were stained with H&E (Solarbio, China, Beijing). Images were captured using Pannoramic Scan (3Dhistech, Hungary, Budapest).

### Cell culture

Murine BV2 microglia were provided by Procell (Wuhan, China). Dulbecco's modified Eagle's medium (DMEM, Gibco, USA) was supplied with 10% fetal bovine serum (Gibco) and 1% penicillin-streptomycin (Procell) for maintaining cells in a humid environment containing 5% CO_2_ at 37 °C.

### Cell treatment

Cells were planted into the 12-well plate (1 × 10^5^ cells/well) and stimulated with 1 mM sevoflurane for 12 h. To assess the role of cGAS-STING pathway in sevoflurane-induced inflammation in BV2 cells, we treated cells with the cGAS inhibitor (RU.521, 1 µM, MedChemExpress, USA), the STING inhibitor (H151, 5 µM, MedChemExpress, USA) and the NLRP3 inhibitor (MCC950, 10 µM, MedChemExpress, USA) 30 min before sevoflurane stimulation. To evaluate the role of mitochondrial DNA in sevoflurane-induced inflammation in BV2 cells, we treated cells with the DRP1 inhibitor (Mdivi-1, 100 nM, MedChemExpress), the mPTP inhibitor (CsA, 1 µM, TargetMol, China) or the VDAC inhibitor (VBIT-4, 10 µM, TargetMol) 30 min before sevoflurane stimulation.

### Preparation of sevoflurane solutions

Stock solutions of the volatile anaesthetics dissolved in the medium were prepared using a modified Masaya-Kudo method 37. A 10 mM stock solution was prepared by injecting 70 µL liquid sevoflurane into 500 µL DMSO and then into 50 mL DMEM 38, 39. The concentration of sevoflurane was diluted to 1 mM prior to use, which the study reported to be a clinically relevant inhalation concentration of 4% in the gas phase 40.

### Western blot

Hippocampal or cell proteins were extracted using RIPA lysate (containing protease inhibitors, Solarbio, China). Samples were completely disrupted at 4 °C for 30 min, followed by centrifugation at 13,000 g for 15 min, and the determination of total protein concentration by bicinchoninic acid assay (BCA, Solarbio). After density at 95°C for 10 min, 30 μg of protein was loaded onto an SDS-PAGE gel for subsequent electrophoresis. Western blotting was performed as previously described 11, 41, 42. ChemiDoc XRS (Bio-Rad, USA) was used to visualise immunoreactive proteins. The relative intensity of the bands was quantified using the Image Lab Analyzer software (Bio-Rad). The antibodies used in this study are listed in Table 1.

### Real-time PCR

Total RNA was extracted and purified from mouse hippocampus or cells using the RNAiso Plus kit (Takara, Kusatsu, Japan). RNA was then reverse transcribed into cDNA. Real-time PCR was performed on a CFX96 Touch™ instrument. Relative expression of genes was calculated by the 2^-ΔΔCt^ method according to our previous study 10, 43, 44. The primer sequences used in the study are shown in Table 2.

### Immunofluorescence

Brain slides were deparaffinized and placed in 0.01 M citrate buffer (pH 6.0). An autoclave was used to heat the citric acid buffer and keep it boiling for 2 min. Then, slides were endogenous peroxidase blocked with 3% H_2_O_2_ and incubated in 5% albumin bovine V (Solarbio) for 1 h. Subsequently, sections were incubated with the primary antibodies at 4 °C overnight, including IBA1 antibody (1:800, Proteintech, Wuhan, China), p-DRP1 (1:200, CST, USA), ASC (1:200, CST), and cGAS (1:200, CST). The next day, after washing with phosphate-buffered saline (PBS) buffer, the Tyramide signal amplification kit (AiFang biological, Hunan, China) was used for fluorescent double-label staining. Nuclei were counterstained with DAPI (Invitrogen, USA) for 1 min. Images were taken with a Pannoramic Scan (3Dhistech, Hungary, Budapest).

The cells were washed with PBS and fixed with 4% paraformaldehyde for 15 min. The cells were permeated with 0.1% Triton X-100 and blocked in 1% BSA for 30 min before being incubated with an anti-rabbit TOM20 antibody (1:200, Proteintech) at 4 °C overnight. After washing with PBS, the sections were placed at room temperature with FITC goat anti-rabbit IgG (ABclonal, China) for 1 h and counterstained with DAPI for 1 min. Images were acquired with a Laser Scanning Confocal Microscope (Leica SP8, Germany).

### Measurement of mitochondrial membrane potential

The mitochondrial membrane potential was measured using the JC-1 Assay Kit (Beyotime Institute of Biotechnology, Shanghai, China). After washing with PBS, the cells were incubated with Double dilution of JC-1 working solution using DMEM medium at 37 ℃ for 20 min. Images were obtained using a fluorescence microscope. The red/green immunosignals were analyzed using Image J software.

### Measurement of cytosolic mtDNA

As described previously 24, mtDNA was extracted from BV2 cells and measured by Real-time PCR analysis. For the detection of mtDNA in the cytosol, BV2 cells were seeded at 6-well plates with a density of 10^6^ cells/well. Cells were incubated with 1% NP-40 on ice for 20 min. The supernatants were collected and centrifuged at 16,000 g for 15 min at 4°C. The cytosolic fractions were collected to extract genomic DNA following the instructions of the QIAamp® DNA Mini Kit (QIAamp GmbH, Hilden, German). Quantification of mitochondrial DNA was performed by Real-time PCR analysis using primers specific for Mitochondrial cytochrome b (Cytb), NADH Dehydrogenase 1 (ND1), and Mitochondrial displacement loop (D-loop).

### Statistical analysis

All data were presented as means ± standard deviation and analyzed with GraphPad Prism 8 software (San Diego, CA, USA). An unpaired *t*-test was used for statistical comparisons between the two groups. ANOVA was used to determine differences between multiple groups, followed by Bonferroni correction for multiple comparisons. All experiments were independently repeated three times. *P<0.05* was considered statistically significant.

## Results

### Prolonged anesthesia with sevoflurane triggers cognitive dysfunction and neurological impairs in mice

The schedule of the animals' experimental procedure is summarized in Figure 1A. In this study, a cognitive dysfunction mouse model was induced by sevoflurane exposure, and cognitive function was assessed after 24 h. The passive avoidance test consists of a dark avoidance test and a shuttle experiment. In the dark avoidance test, the number of entries into the darkroom in the POCD group was increased compared with the control group (*P*<0.01), and the avoiding the dark latency period decreased in the POCD group (*P*<0.001, Figure 1B-C). In the shuttle experiment, compared with the control group, the active avoidance times of mice in the POCD group decreased (*P*<0.001, Figure 1D). We then used the Y-maze to assess spatial learning and memory in mice. In the training phase of the Y-maze test, mice in the POCD group covered fewer total distances. They had a longer total resting time than the control group (*P*<0.01), indicating that prolonged anesthesia may reduce the desire of mice to explore new environments (*P*<0.05, Figure 1E-G). For the test phase of the Y-maze test, the percentage of spontaneous changes was lower in the POCD group than in the control group (*P*<0.05, Figure 1H-I). Moreover, we also found that the POCD group mice had a decreased number of entries into the novel arm compared with the control group (*P*<0.05, Figure 1J). There was no significant difference in the duration spent in the novel arm between the control and POCD groups (Figure 1K). This evidence suggests that prolonged anesthesia triggers cognitive dysfunction.

Our subsequent attention turned to the effects of sevoflurane exposure on neurons, the executive cellular basis of cognitive function. H&E staining showed no noticeable pathological changes in neurons in the hippocampal region of the control group. However, in the DG, CA1, and CA3 regions of the hippocampus, the brain tissue of mice in the POCD group showed a disorganized arrangement and a decrease in the number of neurons (Figure 1L). We further measured the levels of amyloid precursor protein (APP), Tau, and p-Tau^T231^, closely associated with cognitive dysfunction, in the hippocampal tissue of mice. We found that the levels of APP and p-Tau^T231^ were upregulated in the POCD group compared to the control group, and there was no difference in the protein level of Tau (both *P*<0.05, Figure 1M-N). Together, these data illustrate that prolonged sevoflurane anesthesia impairs the learning and memory ability of mice.

### Sevoflurane induces the microglial NLRP3 inflammasome activation in POCD mice

**Figure 2:**
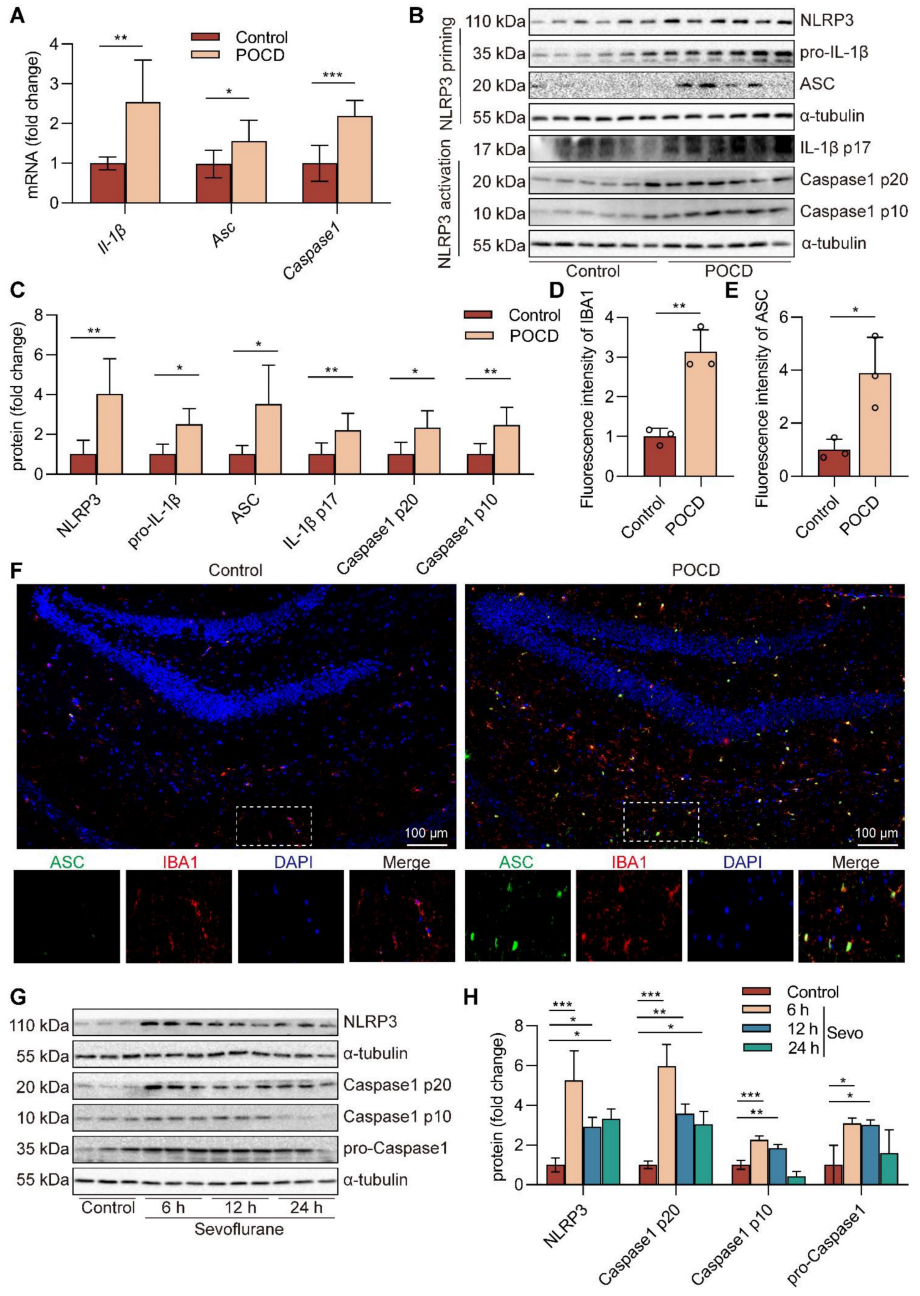
** Sevoflurane induces the microglial NLRP3 inflammasome activation in POCD mice. A** *Il-1β*, *Asc*, and *Caspase1* mRNA expressions in the hippocampus were detected by real-time PCR (*n*=6). **B-C** The protein expressions of NLRP3, pro-IL-1β, ASC, IL-1β p17, Caspase1 p20, and Caspase1 p10 in the hippocampus was detected by western blot (*n*=6). **D-F** The fluorescence intensity of ASC (green) and IBA1 (red) in the hippocampal was detected by immunofluorescence (*n*=3, bar=100 µm). **G-H** BV2 cells were treated with 1 mM sevoflurane for 6, 12, and 24 h. The protein expression of NLRP3, Caspase1 p20, Caspase1 p10, and pro-Caspase1 in the BV2 cells was detected by western blot (*n*=3). Data are expressed as the mean ± SD. Comparisons between the two groups were made with an unpaired *t*-test. Differences among multiple groups were performed using ANOVA. *P < 0.05, **P < 0.01, and ***P < 0.001.

Neuroinflammation is an indispensable mechanism of cognitive dysfunction 33, and NLRP3 inflammasome is a research hotspot for neuroinflammation 45. After sevoflurane exposure, NLRP3 inflammasome components, including *Il-1β* mRNA (*P*<0.01)*, Asc* mRNA (*P*<0.05)*,* and* Caspase1* mRNA (*P*<0.001) in the hippocampus, were upregulated compared with those in the control group (Figure 2A). Besides, the protein expressions of NLRP3 (*P*<0.01), pro-IL-1β (*P*<0.05), ASC (*P*<0.05), IL-1β p17 (*P*<0.01), Caspase1 p20 (*P*<0.05), and Caspase1 p10 (*P*<0.01) in hippocampal tissue of mice were markedly increased in the POCD group (Figure 2B-C). These data suggest that NLRP3 inflammasome is activated in hippocampal tissue of sevoflurane-anaesthetized mice. Microglia are resident phagocytes in the central nervous system and essential effector cells in neuroinflammation 5. Then, we found that the hippocampus of the mice expressed high levels of the microglial activation markers IBA-1 and ASC protein after exposure to sevoflurane. In addition, more activated IBA-1^+^ microglia co-localized with ASC protein in the POCD group (Figure 2D-F). To evaluate if sevoflurane led to activation of the NLRP3 inflammasome in microglia, we treated BV2 microglia with sevoflurane for different points in time. The results showed that the levels of NLRP3 (*P*<0.05), Caspase1 p20 (*P*<0.01), Caspase1 p10 (*P*<0.01), and pro-Caspase1 (*P*<0.05) proteins were significantly increased in BV2 microglia after sevoflurane treatment for 12 h (Figure 2G-H). Meanwhile, the levels of pro-IL-1β (*P*<0.01) and IL-1β p17 (*P*<0.001) proteins were significantly increased in BV2 microglia after sevoflurane treatment for 12 h (Figure S1). These data indicate that sevoflurane induces the NLRP3 inflammasome activation of microglial in the POCD mice.

### Sevoflurane activates the cGAS-STING pathway in POCD mice

**Figure 3:**
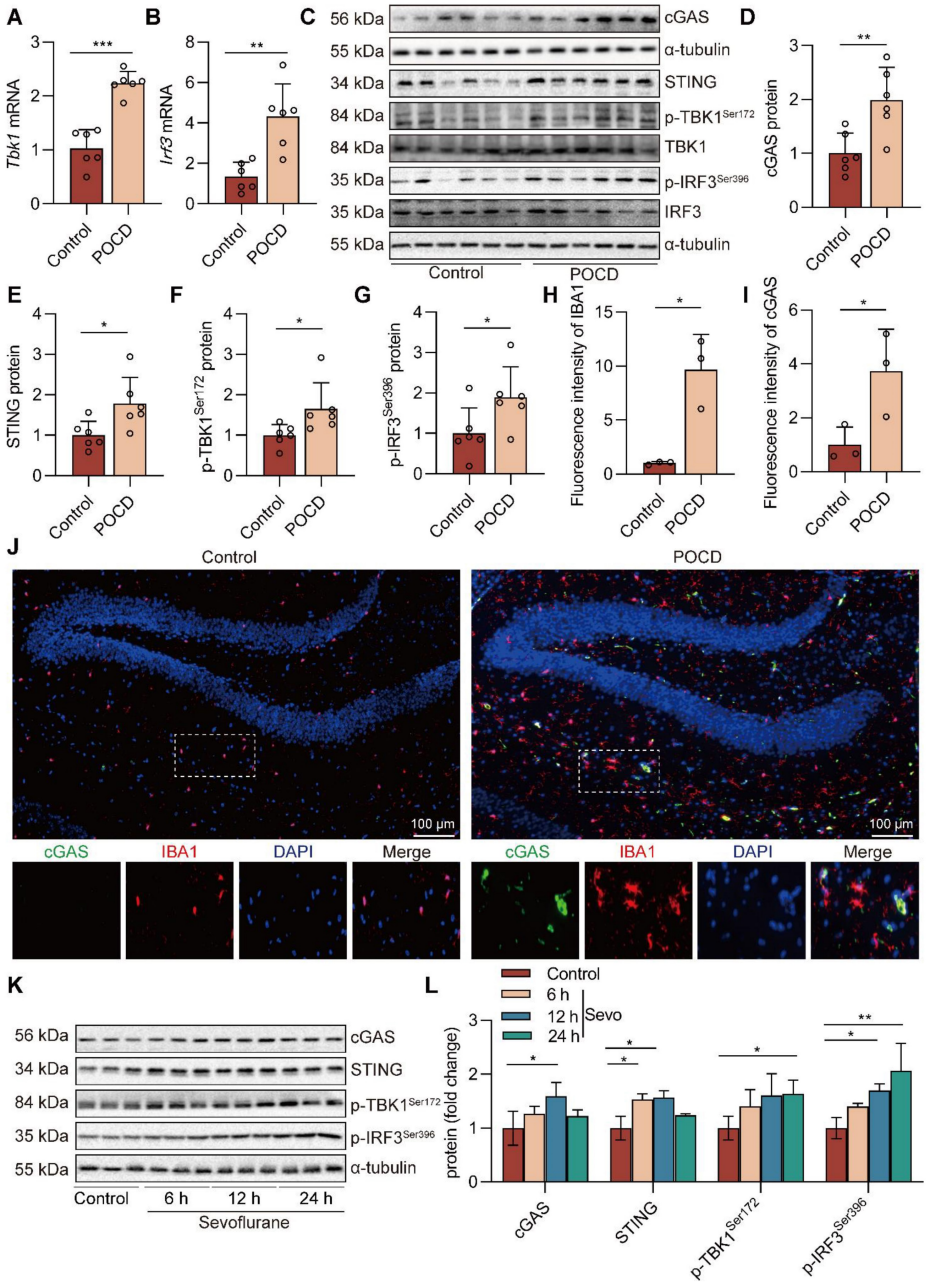
** Sevoflurane activates the cGAS-STING pathway in POCD mice. A-B** *Tbk1* and *Irf3* mRNA expression in the hippocampus were detected by real-time PCR (*n*=6). **C-G** Protein levels of cGAS, TBK1, STING, p-TBK1^Ser172^, p-IRF3^Ser396^, and IRF3 in the hippocampus were detected by western blot (*n*=6). **H-J** The fluorescence intensity of cGAS (green) and IBA1 (red) in hippocampal tissue was detected by immunofluorescence (*n*=3, bar=100 µm). **K-L** Protein levels of cGAS, STING, p-TBK1^Ser172^, and p-IRF3^Ser396^ in the BV2 cells were detected by western blot (*n*=3). Data are expressed as the mean ± SD. Comparisons between the two groups were made with an unpaired *t*-test. Differences among multiple groups were performed using ANOVA. *P < 0.05, **P < 0.01, and ***P < 0.001.

cGAS has been identified as a critical cytosolic DNA sensor closely associated with animal models of cognitive dysfunction through neuroinflammation 28. Therefore, we next establish the effects of prolonged anesthesia on hippocampal cGAS-STING pathway gene and protein expression. We observed that sevoflurane exposure increased cGAS-STING pathway *Tbk1* (*P*<0.001) and *Irf3* (*P*<0.01) gene expression in the hippocampus (Figure 3A-B). The levels of cGAS (*P*<0.01), STING (*P*<0.05), p-TBK1^Ser172^ (*P*<0.05), and p-IRF3^Ser396^ (*P*<0.05) in the hippocampal tissue of mice were significantly increased in the POCD group (Figure 3C-G). Meanwhile, immunofluorescence double staining showed that cGAS, at high levels after sevoflurane exposure, was mainly expressed in IBA-1^+^ microglia in the hippocampus (Figure 3H-J). We also treated BV2 microglia with sevoflurane. After 6, 12, and 24 h, cGAS-STING pathway protein expression was detected by western blot. The results showed that the levels of cGAS (*P*<0.05), STING (*P*<0.05), p-TBK1^Ser172^ (*P*<0.05), and p-IRF3^Ser396^ (*P*<0.01) were increased in BV2 microglia treated with sevoflurane at 12 h (Figure 3K-L). Taken together, these data illustrate that sevoflurane triggers the activation of the microglial cGAS-STING pathway in the POCD mice.

### Inhibition of cGAS decreases cognitive dysfunction in POCD mice

**Figure 4:**
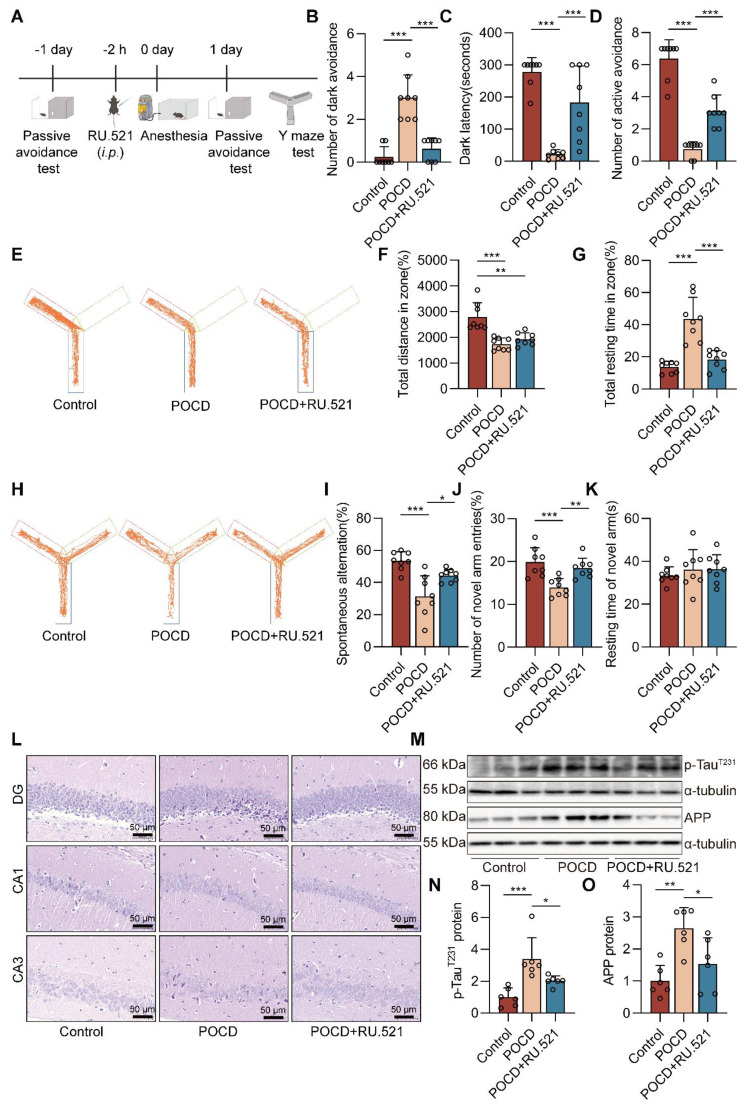
** Inhibition of cGAS decreases cognitive dysfunction in POCD mice. A** C57BL/6J mice were trained in the passive avoidance test. The next day, the mouse was given intraperitoneal injection of RU.521 (5 mg/kg) before 2 h. Then, 4% sevoflurane was exposed to mice for 6 h to induce cognitive impairment. On the first day after sevoflurane exposure, the mice underwent the passive avoidance test and the Y-maze test. **B-C** The dark avoidance test was conducted to assess the number of entering the darkroom and the dark latency (*n*=8). **D** The shuttle experiment was conducted to assess active avoidance (*n*=8).** E** Representative trajectories of each group in the training session of the Y-maze test. **F-G** Total distances and total resting times were assessed in the Y-maze training (*n*=8). **H** Representative trajectories of each group in the Y-maze testing. **I-K** The percentage of spontaneous alternation, the number of entries into the novel arm, and the duration spent in the novel arm were assessed in the Y-maze testing (*n*=8). **L** HE staining of hippocampal tissue (bar=50 µm). **M-N** Protein levels of p-Tau^T231^ and APP in the hippocampus were detected by western blot (*n*=6). Data are expressed as the mean ± SD. Differences among multiple groups were performed using ANOVA. *P < 0.05, **P < 0.01, and ***P < 0.001*.*

To investigate the role of the cGAS-STING pathway in cognitive function in sevoflurane exposed mice, we used RU.521, an inhibitor of cGAS. The experimental procedure for the animals is summarized in Figure 4A. In the dark avoidance test, the RU.521 treatment effectively reduced the number of mice entering the dark room and increased the dark avoidance latency (both *P*<0.001, Figure 4B-C). In the shuttle experiment, RU.521 treatment increased the active avoidance times of mice, indicating that RU.521 significantly attenuates sevoflurane-induced memory loss in POCD mice (*P*<0.001, Figure 4D). In the Y-maze test, we also found that RU.521 treatment reduced total mouse resting time (*P*<0.001, Figure 4G) and increased the percentage of spontaneous changes (*P*<0.05 vs. POCD group, Figure 4H-I) and the number of new arm entries (Figure 4J).

RU.521 had no significant effect on mouse total distances traveled (Figure 4E-F) and time spent in the novel arm (Figure 4K). To further clarify the effects of RU.521 treatment on motor and cognitive function in POCD mice, we also used the Open field test and the Elevated plus maze. In the Open field test, RU.521 treatment increases the POCD mice spend in the residence time in the central area (*P*<0.01 vs. POCD group, Figure S2). In the Elevated plus maze, treatment with RU.521 increases the ratio of the time spent in the open arm (*P*<0.001 vs. POCD group) and the ratio of the times spent entering the open arm in POCD mice (*P*<0.05 vs. POCD group, Figure S2). It illustrated that RU.521 treatment could reverse the spatial learning and memory deficit induced by sevoflurane. HE staining showed that RU.521 attenuated neuron damage in the hippocampal region of sevoflurane-treated mice (Figure 4L). Western blot analysis showed that the levels of APP and p-Tau^T231^ proteins were reduced in the hippocampus of the mice after treatment with RU.521 (both *P*<0.05 vs. POCD group, Figure 4M-O). Overall, these results indicate that inhibition of cGAS-STING pathway activation could alleviate cognitive dysfunction in POCD mice.

### Inhibition of cGAS suppresses the NLRP3 inflammasome-mediated neuroinflammation in POCD mice

**Figure 5:**
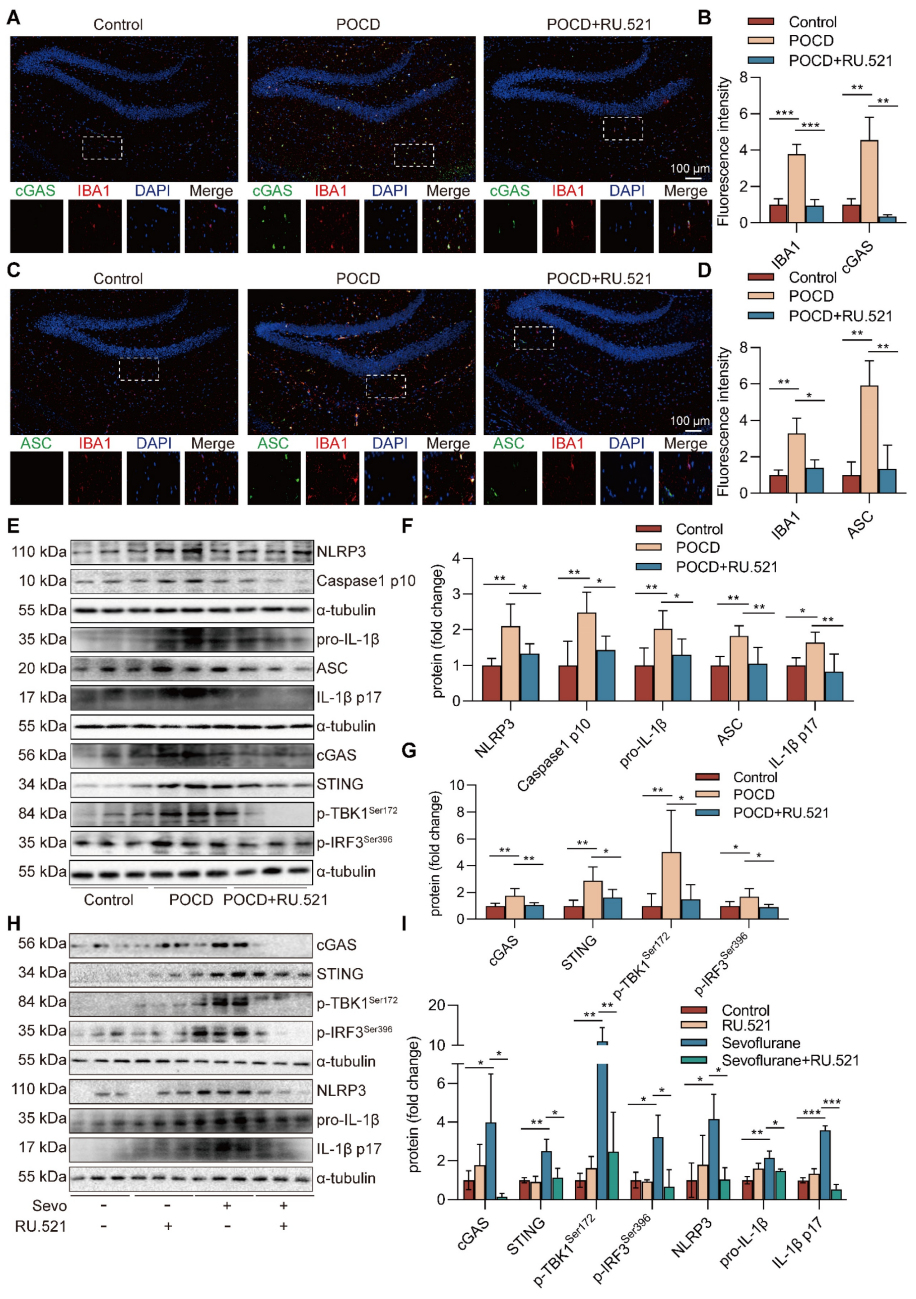
** Inhibition of cGAS suppresses the NLRP3 inflammasome-mediated neuroinflammation in POCD mice. A-B** The fluorescence intensity of cGAS (green) and IBA1 (red) in hippocampal tissue was detected by immunofluorescence (*n*=3, bar=100 µm). **C-D** The fluorescence intensity of ASC (green) and IBA1 (red) in hippocampal tissue was detected by immunofluorescence (*n*=3, bar=100 µm). **E-G** Protein levels of NLRP3, pro-IL-1β, ASC, IL-1β p17, Caspase1 p10, cGAS, STING, p-TBK1^Ser172^, and p-IRF3^Ser396^ in the hippocampus were detected by western blot (*n*=6). **H-I** BV2 cells were treated with 1 mM sevoflurane for 12 h after 1 µM RU.521 intervention 30 min. Protein levels of cGAS, STING, p-TBK1^Ser172^, p-IRF3^Ser396^, NLRP3, pro-IL-1β, and IL-1β p17 in the BV2 cells were detected by western blot (*n*=3). Data are expressed as the mean ± SD. Differences among multiple groups were performed using ANOVA. *P < 0.05, **P < 0.01, and ***P < 0.001.

When there is an infection, cellular damage, or tissue injury, the cGAS-STING signaling pathway becomes an essential link in inflammation 22. Whether the cGAS-STING pathway can be a trigger for NLRP3 inflammasome activation in sevoflurane-treated mice is unclear. Herein, we used immunofluorescence double staining to detect the effects of RU.521 treatment on the expression of ASC, cGAS, and IBA1 proteins in the mouse hippocampus. The results showed that the RU.521 treatment reduced the protein expressions of ASC, cGAS, and IBA1 in the mouse hippocampus (Figure 5A-D). Real-time PCR results show that inhibition of cGAS reduces the levels of a number of genes downstream of the cGAS-STING pathway in hippocampal tissue from POCD mice (*P*<0.05 or *P*<0.01 vs. POCD group, Figure S6). Meanwhile, western blot results showed that after RU.521 treatment, the levels of NLRP3 inflammasome-related protein (NLRP3, pro-IL-1β, ASC, IL-1β p17 and Caspase1 p10) and cGAS-STING pathway-related protein (cGAS, STING, p-TBK1^Ser172^, and p-IRF3^Ser396^) were decreased in the mouse hippocampus (*P*<0.05 or *P*<0.01 vs. POCD group, Figure 5E-G). To further demonstrate that RU.521 could reduce sevoflurane-induced NLRP3 inflammasome activation at the cellular level, we pre-treated BV2 microglia with RU.521. Using immunofluorescence, we observed that inhibition of cGAS reduced NLRP3 and ASC co-localization and expression in sevoflurane-treated BV2 cells, as well as the fluorescence intensity of pro-caspase1 protein (Figure S5). RU.521 also alleviated the NLRP3 inflammasome-related proteins (NLRP3, pro-IL-1β, and IL-1β p17) and the cGAS-STING pathway-related proteins (cGAS, STING, p-TBK1^Ser172^, and p-IRF3^Ser396^) induced by sevoflurane (Figure 5H-I). We additionally pre-treated BV2 cells with a STING inhibitor (H151) and NLRP3 inhibitor (MCC950). The results showed that inhibition of STING reduced NLRP3 inflammasome activation (*P*<0.05 or *P*<0.01 vs. Sevo group, Figure S4), whereas inhibition of NLRP3 did not affect the cGAS-STING pathway activation (*P*>0.05 vs. Sevo group, Figure S3). Collectively, these data illustrate that sevoflurane can result in the cGAS-STING pathway-dependent NLRP3 inflammasome activation in the microglia of POCD mice.

### Sevoflurane triggers mitochondrial fission and causes mtDNA escape into the cytosol in microglia

**Figure 6:**
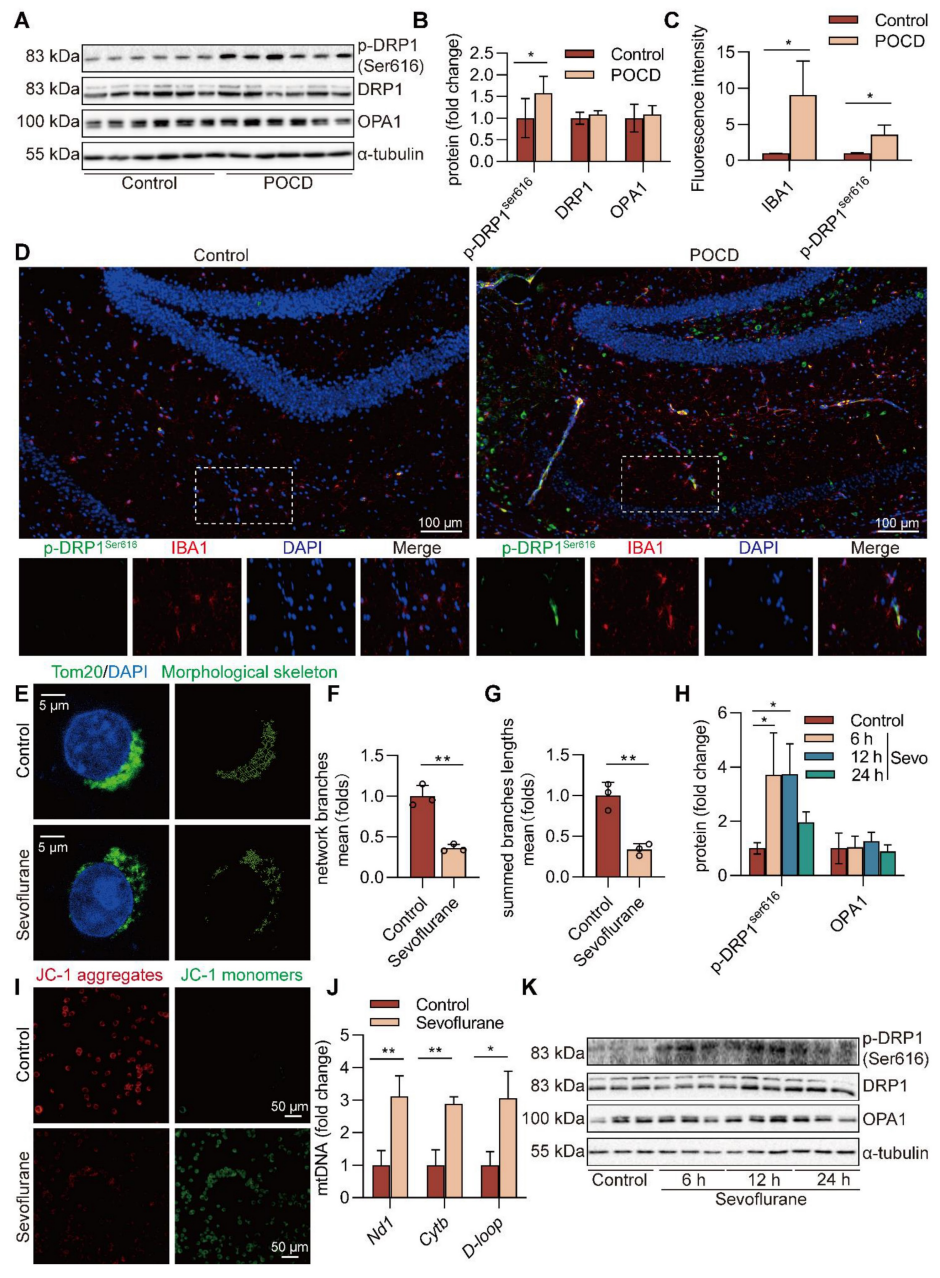
** Sevoflurane triggers mitochondrial fission and causes mtDNA escape into the cytosol in microglia. A-B** Protein levels of p-DRP1^Ser616^, DRP1, and OPA1 in the hippocampus were detected by western blot (*n*=6). **C-D** The fluorescence intensity of p-DRP1^Ser616^ (green) and IBA1 (red) in the hippocampal was detected by immunofluorescence (*n*=3, bar=100 µm). **E** Mitochondrial morphology stained with TOM20 in BV2 cells (bar=5 µm). **F-G** The morphological skeleton and quantification of mitochondrial morphology were detected in BV2 cells (*n*=3). **I** Representative images of BV2 cells loaded with the mitochondrial membrane potential indicator JC-1 (bar=50 μm). **J** mtDNA levels of *Nd1, Cytb,* and *D-loop* in the cytoplasm of BV2 cells were determined by real-time PCR (*n*=3). **H, K** Protein levels of p-DRP1^Ser616^, DRP1, and OPA1 in the BV2 cells were detected by western blot (*n*=3). Data are expressed as the mean ± SD. Comparisons between the two groups were made with an unpaired *t*-test. Differences among multiple groups were performed using ANOVA. *P < 0.05 and **P < 0.01*.*

Mitochondria, which play a pivotal role in inflammation, are highly dynamic organelles that undergo constant fission and fusion, and these morphological changes affect the state of the cell. We found no difference in the mitochondrial fusion protein optic atrophy 1 (OPA1) levels between health and sevoflurane-induced POCD mice. However, the level of p-DRP1^Ser616^ (*P*<0.05), which triggers mitochondrial fission, was increased after sevoflurane exposure in mice (Figure 6A-B). The immunofluorescence double staining also showed that p-DRP1^Ser616^, at high levels after sevoflurane exposure, was mainly expressed in IBA-1^+^ microglia in the mouse hippocampus (Figure 6C-D). These results suggest that sevoflurane may induce mitochondrial fission in the microglia of POCD mice.

To further determine the mitochondrial function changes in sevoflurane-treated microglia, we measured mitochondrial membrane potential (MMP) at the cellular level, an index of mitochondrial function. This result showed that MMP was dissipated by the treatment with sevoflurane (Figure 6I), indicating that sevoflurane exacerbates mitochondrial dysfunction of microglia.

In addition, we characterized the morphology of the mitochondrial network by immunocytochemistry, using an antibody against the outer mitochondrial membrane protein TOM20. A significant fragmentation was found in sevoflurane-treated microglia (*P*<0.01, Figure 6E-G). In particular, the level of p-DRP1^Ser616^ was highly induced in microglia treated with sevoflurane after 6 and 12 h (*P*<0.05, Figure 6H, K). mtDNA can easily be released from damaged mitochondria to cytosol under mitochondrial fission. To confirm whether mtDNA escaped into the cytosol, we extracted the DNA in the cytosol and performed real-time PCR amplification. The results showed that the mtDNA in the sevoflurane group was significantly enriched compared with the control group (*P*<0.05 or *P*<0.01, Figure 6J). These data imply that sevoflurane induces mitochondrial fission and subsequent release of mtDNA into the cytoplasm in microglia.

### Blockade of DRP1 reduces sevoflurane-induced cGAS-STING pathway-dependent NLRP3 inflammasome in microglia

**Figure 7:**
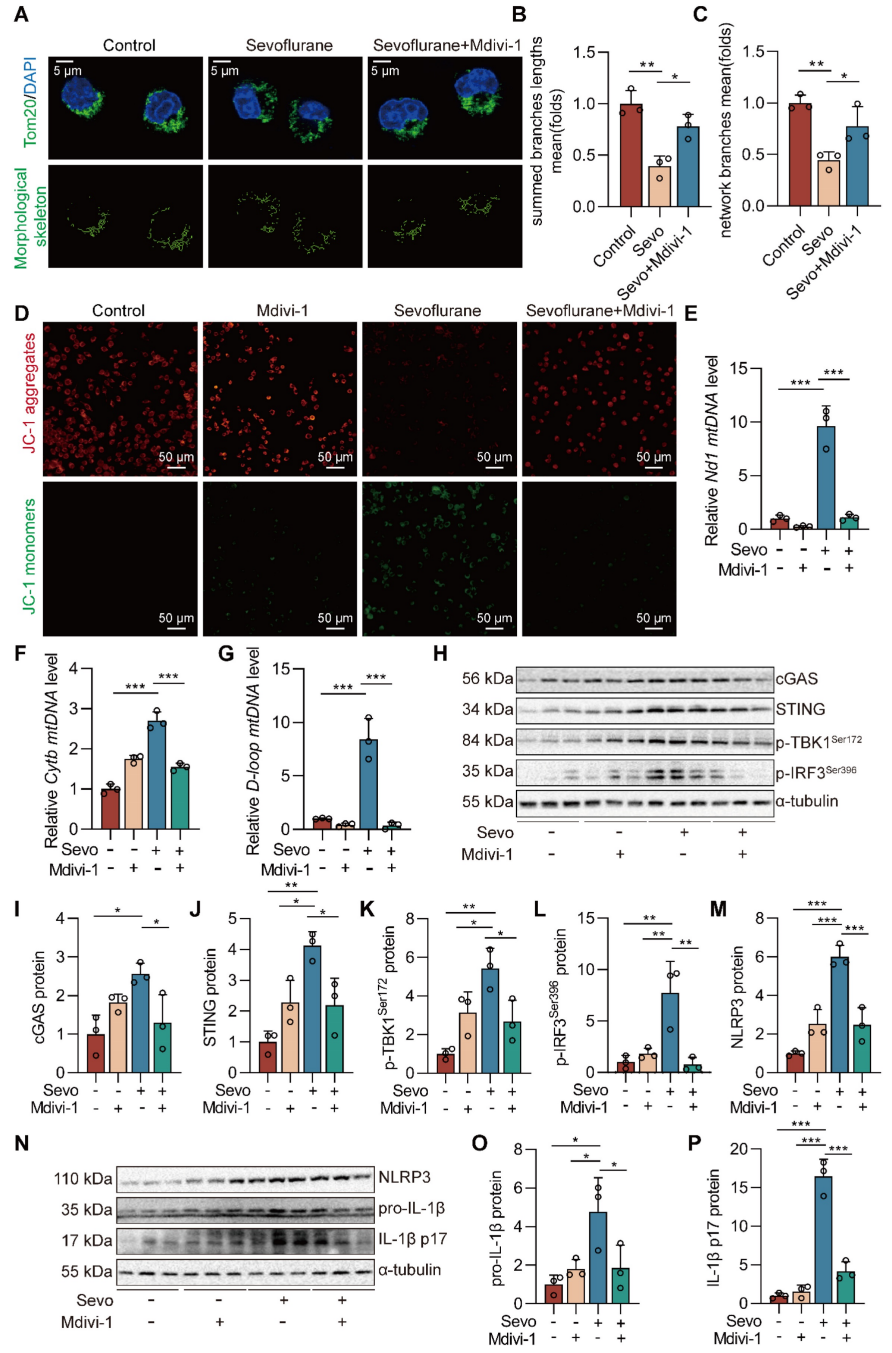
** Blockade of DRP1 reduces sevoflurane-induced cGAS-STING pathway-dependent NLRP3 inflammasome in microglia. A** BV2 cells were treated with 1 mM sevoflurane for 12 h after 100 nM Mdivi-1 intervention 30 min. Mitochondrial morphology stained with TOM20 in BV2 cells (bar=5 µm). **B-C** The morphological skeleton and quantification of mitochondrial morphology were detected in BV2 cells (*n*=3). **D** Representative images of BV2 cells loaded with the mitochondrial membrane potential indicator JC-1 (bar=50 μm). **E-G** mtDNA levels of *Nd1, Cytb,* and *D-loop* in the cytoplasm of BV2 cells were determined by real-time PCR (*n*=3). **H-L** Protein levels of cGAS, STING, p-TBK1^Ser172^, and p-IRF3^Ser396^ in the BV2 cells were detected by western blot (*n*=3).** M-P** The protein levels of NLRP3, pro-IL-1β, and IL-1β p17 in the BV2 cells were detected by western blot (*n*=3). Data are expressed as the mean ± SD. Differences among multiple groups were performed using ANOVA. *P < 0.05, **P < 0.01, and ***P < 0.001*.*

The cGAS-STING pathway can be activated by dsDNA and participates in the occurrence and development of inflammatory diseases 24. We further investigated whether sevoflurane-induced mitochondrial fission was necessary for the activation of the cGAS-STING pathway in microglia and pre-treated BV2 microglia with Mdivi-1, an inhibitor of DRP1. First, Mdivi-1 reversed mitochondrial fragmentation induced by sevoflurane in microglia (both *P*<0.05 vs. Sevo group, Figure 7A-C). Then, we observed that Mdivi-1 reduced the sevoflurane-induced loss of MMP in microglia (Figure 7D). Mdivi-1 also reduced the release of mtDNA into the cytoplasm in microglia treated with sevoflurane (both *P*<0.001 vs. Sevo group, Figure 7E-G). The pharmacological inhibition of DRP1 significantly reduced the level of cGAS-STING pathway-related proteins (cGAS, STING, p-TBK1^Ser172^, and p-IRF3^Ser396^) by sevoflurane treatment in microglia (*P*<0.05 or *P*<0.01 vs. Sevo group, Figure 7H-L). Finally, we found that Mdivi-1 decreased the expression of the NLRP3 inflammasome-related proteins (NLRP3, pro-IL-1β, and IL-1β p17) induced by sevoflurane (*P*<0.05 or *P*<0.001 vs. Sevo group, Figure 7M-P). These results imply that mitochondrial fission is necessary for sevoflurane-induced activation of the NLRP3 inflammasome* via* the cGAS-STING pathway in microglia.

### Inhibiting mPTP-VDAC channel opening attenuates sevoflurane-induced mtDNA cytosolic escape and reduces cGAS-STING pathway activation in microglia

**Figure 8:**
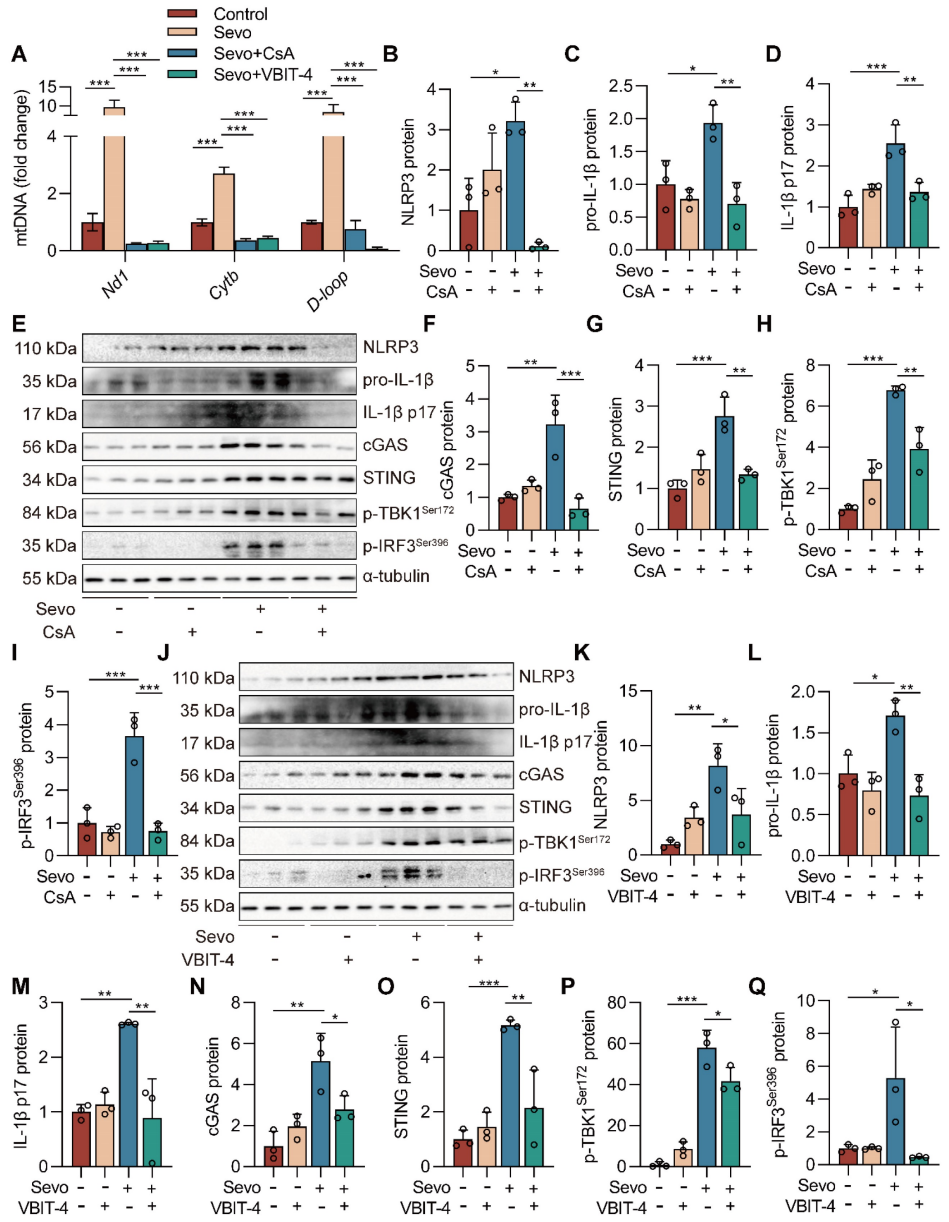
**Inhibiting mPTP-VDAC channel opening attenuates sevoflurane-induced mtDNA cytosolic escape and reduces cGAS-STING pathway activation in microglia.** A BV2 cells were treated with 1 mM sevoflurane for 12 h after 1 µM CsA intervention for 30 min and 10 µM VBIT-4 intervention for 30 min. mtDNA levels of *Nd1, Cytb,* and *D-loop* in cytoplasm of BV2 cells were determined by real-time PCR (*n*=3). B-I Protein levels of NLRP3, pro-IL-1β, IL-1β p17, cGAS, STING, p-TBK1^Ser172^, and p-IRF3^Ser396^ in the BV2 cells were detected by western blot (*n*=3). J-Q Protein levels of NLRP3, pro-IL-1β, IL-1β p17, cGAS, STING, p-TBK1^Ser172^, and p-IRF3^Ser396^ in the BV2 cells were detected by western blot (*n*=3). Data are expressed as the mean ± SD. Differences among multiple groups were performed using ANOVA. *P < 0.05, **P < 0.01, and ***P < 0.001.

The mPTP-VDAC channel, closely associated with mitochondrial morphology and function, can allow mtDNA to pass under cell injury 27, 28. To investigate the role of the mPTP-VDAC channel in the sevoflurane-induced release of mtDNA into the cytoplasm, we used the mPTP inhibitor cyclosporine A (CsA) and the VDAC oligomerization inhibitor VBIT-4 to pre-treat the microglia. Pre-treating microglia with CsA and VBIT-4 significantly attenuated sevoflurane-induced mtDNA cytosolic escape (both *P*<0.001 vs. Sevo group, Figure 8A). At the same time, western blot results showed that CsA and VBIT-4 treatment decreased the protein expressions of NLRP3 inflammasome-related (NLRP3, pro-IL-1β, and IL-1β p17) and cGAS-STING pathway-related (cGAS, STING, p-TBK1^Ser172^, and p-IRF3^Ser396^) in sevoflurane-treated microglia (*P*<0.05 or *P*<0.001 vs. Sevo group, Figure 8B-Q). These results point to the critical role of the mPTP-VDAC channel opening in the process of the mtDNA release and subsequent the cGAS-STING pathway-dependent NLRP3 inflammasome activation in microglia.

## Discussion

Neuroinflammation with excessively activated microglia has been associated with the pathogenesis and progression of neurodegenerative diseases 46. Increased experimental evidence has established that microglia-mediated inflammation is essential in postoperative cognitive dysfunction 47, 48. In this study, we demonstrated that sevoflurane induced the activation of the cGAS-STING pathway, which triggered NLRP3 inflammasome-associated neuroinflammation in microglia during POCD. Mechanically, we identified that sevoflurane-induced mitochondrial fission-mediated release of mtDNA and then activated the cGAS-STING pathway-dependent NLRP3 inflammasome of microglia. In conclusion, our study provides new insights into the molecular mechanisms associated with the cGAS-STING pathway and its role as a neuroinflammatory pathology in POCD.

Patients with cognitive decline following anaesthesia/surgery have been the focus of attention since 1955 49. In 2018, the Nomenclature Consensus Working Group recommended "PND" as an umbrella term for preoperative or postoperative findings of cognitive impairment, which encompasses preoperatively diagnosed cognitive decline, POD, delayed neurocognitive recovery, and POCD 50. Given that we were focusing on cognitive changes during the perioperative period, we chose to use the term "POCD" in the current study. General anesthetics are indispensable for surgical procedures in preclinical and clinical work. Sevoflurane is one of the most widely used anesthetics in preclinical and clinical settings.

However, prolonged sevoflurane anaesthesia or exposure to a high dose of sevoflurane can trigger sevoflurane-induced neurotoxicity 29, 51. Sevoflurane induces cognitive impairment in mice *via* the ROS-NLRP3 inflammasome pathway and also causes pyroptosis in rat hippocampal neurons, leading to cognitive decline 25, 30. Interestingly, we also found that prolonged high-dose sevoflurane anaesthesia induced hippocampus-dependent cognitive decline by the passive avoidance test and Y-maze test in mice. POCD is phenotypically similar to neurocognitive diagnoses in the Diagnostic and Statistical Manual of Mental Disorders, such as Alzheimer's Disease (AD) 50. After sevoflurane anaesthesia, the early AD event that exacerbates amyloid-beta deposition and Tau-induced neurodegeneration occurs in the mouse hippocampus, which is consistent with our findings 52, 53. Therefore, these studies suggest that sevoflurane-induced neurotoxicity is closely associated with postoperative cognitive dysfunction.

Our study clarified a novel mechanism of NLRP3 inflammasome activation-mediated inflammation in microglia during POCD. The NLRP3 inflammasome plays a coordinative role in determining the intensity of microglia activation under different pathological conditions. In addition, it has been reported that neuroinflammatory response mediated by the NLRP3 inflammasome was involved in the pathological process of POCD 54. Using NLPR3-knockout mice and an NLRP3 inhibitor, tissue inflammatory response was significantly inhibited, causing amelioration of murine cognitive dysfunction 55. Our study also supported NLRP3 inflammasome activation in mouse hippocampal microglia after sevoflurane exposure, leading to a deterioration in cognitive function. While its induction mechanism has not been thoroughly elucidated. There is increasing evidence that the cGAS-STING pathway is implicated in NLRP3-mediated neuroinflammation, contributing to neurodegenerative disease progression. The NLRP3 inflammasome activation requires two steps: priming and activation. Stimulating STING triggers the activation of the transcription factors IRF3 and NF-κB, the latter being a classic signal for priming the NLRP3 inflammasome 56. In addition, another study has shown that the cGAS-STING pathway can cause lysosomal damage and induce K^+^ efflux, which can activate the NLRP3 inflammasome 57. This suggests that cGAS-STING is a signaling pathway essential for the priming and activation of the NLRP3 inflammasome. In our study, we found that the vital DNA sensor protein cGAS expression in the sevoflurane-induced microglia was upregulated, accompanied by phosphorylation and activation of its downstream effector proteins TBK1 and IRF3. RU.521 is the most commonly utilized cGAS inhibitor and has established a neuroprotective role in the brain of an experimental neonatal hypoxia-ischemia rat model by intranasal delivery 58. We found that blocking the cGAS-STING pathway with RU.521 alleviated cognitive dysfunction in sevoflurane-induced mice and reduced NLRP3-mediated inflammation in microglia. However, our next step is to determine how the cGAS-STING pathway triggers NLRP3 inflammasome activation in sevoflurane-induced microglia.

Loss of mitochondrial homeostasis has emerged as a distinct mechanism of the cGAS-STING pathway activation cascade in inflammatory diseases 59. As a critical DNA sensor, cGAS recognizes its own and external DNA to induce the innate immune response 60. Recent studies have shown that the cGAS-STING pathway could detect leaked DNA and activate pro-inflammatory cytokines in mice with experimental chronic kidney disease 61 and also used to activate mtDNA leakage to trigger pathological inflammatory responses in mice with acute pancreatitis 62. We demonstrated that a large amount of mtDNA appeared in the cytoplasm of microglia after sevoflurane treatment, suggesting that mtDNA are thought to be activators of the cGAS-STING pathway in microglia. The mechanism by which mtDNA is released into the cytoplasm remains unclear. Mitochondria are highly dynamic, and balancing mitochondrial fusion and fission is essential for eukaryotic organisms to survive and function. Drp1 is a crucial regulator of mitochondrial fission. DRP1-dependent mitochondrial fission has been proposed to be a prerequisite for mitophagy 26. Here, we found that the mitochondria of sevoflurane-induced microglia exhibited an over-divided morphology and increased levels of Drp1 phosphorylation. Since mitochondrial fission is unequal, the smaller product of peripheral fission often has damaged mtDNA and higher levels of mitoROS. The treatment of Mdivi-1, the DRP1 inhibitor, significantly reduced the release of mtDNA in the cytoplasm and reversed sevoflurane-induced cGAS-STING pathway-dependent NLRP3 inflammasome activation in microglia. Therefore, we speculated that DRP1-dependent mitochondrial fission could activate the cGAS-STING signaling pathway in sevoflurane-induced microglia and cause inflammation by inducing mtDNA escape into the cytosol. Previous studies have demonstrated that mPTP is involved in the VDAC-dependent mtDNA release induced by oxidative stress and pro-inflammatory conditions 24, 63. Next, to further prove that the cGAS-STING signaling pathway could be activated by mtDNA escape from the mPTP-VDAC channel, we introduced CsA (the mPTP inhibitor) and VBIT-4 (the VBIT inhibitor) into microglia. Then, we found that CsA and VBIT-4 effectively inhibited cGAS-STING pathway activation, alleviated NLRP3-mediated inflammation, and reduced cytoplasmic mtDNA release in sevoflurane-induced microglia. DRP1-mediated mitochondrial fission can cause mitochondrial structural disruption, and subsequent over-opening of mPTP and oligomerization of VDAC to exacerbate mitochondrial and cellular dysfunction 27, 28. Therefore, further work is required to determine whether DRP1-mediated mitochondrial fission can mediate the efflux of mtDNA via the mPTP-VDAC channel in sevoflurane-induced microglia.

There are several limitations to this study. First, the current work only focused on cognitive function in mature mice. However, several studies have shown that surgical and anesthetic stress could induce cognitive changes in adult mice. These studies, as with us, are partially responsive to the pathological mechanisms of POCD 64-66. Second, in this study, we have concentrated mainly on early postoperative cognitive function. Therefore, the development of long-term postoperative cognitive function should be further investigated. Finally, to further investigate the involvement of cGAS-STING signaling in NLRP3 inflammasome-dependent neuroinflammation, cGAS or STING knockout mice may be used in our future study.

## Conclusion

**Figure 9:**
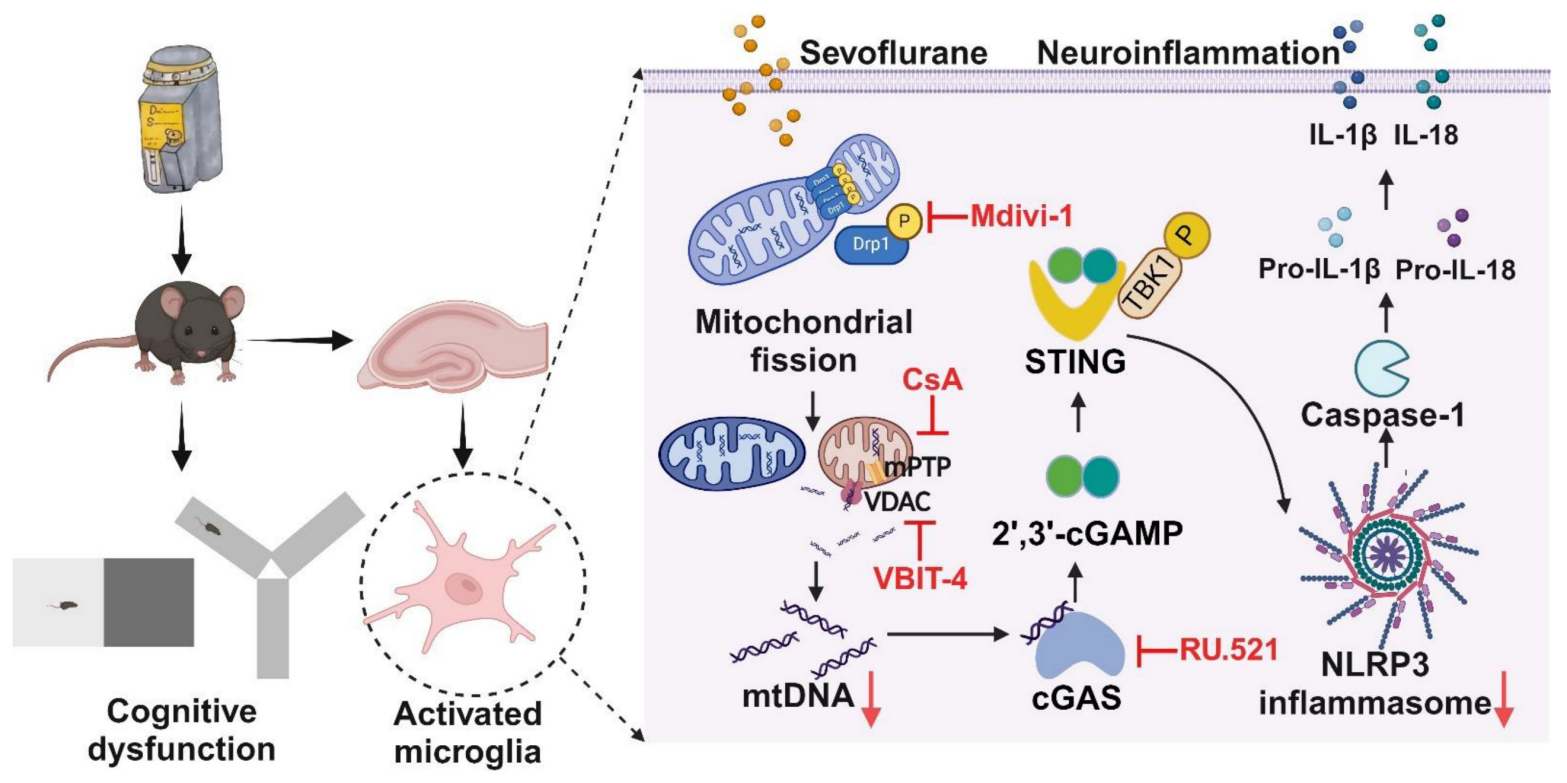
** Schematic illustration.** Sevoflurane promoted DRP1-dependent mitochondrial fission to release mtDNA into the cytoplasm *via* the mPTP-VDAC channel, which induced the cGAS-STING pathway-dependent NLRP3 inflammasome activation, resulting in neuroinflammation of microglia.

In conclusion, our study reveals a crucial role of DRP1-mediated mitochondrial fission in controlling NLRP3 inflammasome activation *via* the cGAS-STING pathway (Figure 9). We suggest that targeting cGAS-STING pathway-dependent NLRP3 inflammasome-associated neuroinflammation in microglia is an effective strategy for treating POCD.

## Supplementary Material

Supplementary figures.

## Figures and Tables

**Table 1:** Antibody sources and dilutions.

Antibody	Source	Catalog	Dilution ratio
**Primary antibodies for western blot**			
Rabbit anti-APP antibody	ABclonal	A17911	1:2000
Rabbit anti-p-Tau^T231^ antibody	ABclonal	AP0053	1:2000
Rabbit-anti-Tau antibody	ABclonal	A19560	1:2000
Rabbit anti-p-IRF3^S396^ antibody	ABclonal	AP0623	1:2000
Rabbit-anti-IRF3 antibody	Proteintech	11312-1-AP	1:2000
Rabbit anti-p-TBK1^S172^ antibody	ABclonal	AP1026	1:2000
Rabbit-anti-TBK1 antibody	Proteintech	28397-1-AP	1:2000
Rabbit-anti-STING antibody	Proteintech	19851-1-AP	1:2000
Rabbit-anti-cGAS antibody	CST	86793SF	1:2000
Rabbit-anti-NLRP3 antibody	CST	15101	1:2000
Rabbit-anti-pro caspase1+p10+P12 antibody	Abcam	Ab179515	1:2000
Rabbit-anti-ASC antibody	CST	67824S	1:2000
Goat-anti-IL-1β polyclonal antibody	R&D	AF-401-NA	1:2000
Rabbit-anti-DRP1 antibody	Abcam	Ab184247	1:2000
Rabbit-anti-p-DRP1^S616^ antibody	CST	3455	1:2000
Rabbit-anti-TOM20 antibody	Proteintech	11802-1-AP	1:2000
Mouse-anti-OPA1 antibody	BD	612606	1:2000
Anti-α-tubulin monoclonal antibody	Servicebio	GB11200	1:10000
**Secondary antibodies for western blot**			
HRP-conjugated goat anti-rabbit IgG	SAB	#L3012-2	1: 5000
Goat anti-Mouse IgG	SAB	L3032	1: 5000
Rabbit anti-Goat IgG	SAB	L3042-2	1: 5000
**Primary antibodies for immunofluorescence**			
Rabbit anti-TOM20 polyclonal antibody	Proteintech	11802-1-AP	1: 200
Rabbit anti-IBA1 antibody	Proteintech	10904-1-AP	1: 800

**Table 2:** Sequences of the primers used in this study.

Gene	Forward primer (5'-3')	Reverse primer (5'-3')
*Caspase1*	CACAGCTCTGGAGATGGTGA	CTTTCAAGCTTGGGCACTTC
*Il-1β*	CAGGCAGGCAGTATCACTCA	AGCTCATATGGGTCCGACAG
*Asc*	GACAGTACCAGGCAGTTCGT	AGTCCTTGCAGGTCAGGTTC
*Tbk1*	GATGTGCTTCACCGAATGGT	CGGCTCGTGACAAAGATAGG
*Irf3*	CTACGGCAGGACGCACAGAT	TCAGCAGCTAACCGCAACAC
*D-loop1*	CCCTTCCCCATTTGGTCT	TGGTTTCACGGAGGATGG
*Cytb*	GCTTTCCACTTCATCTTACCATTTA	TGTTGGGTTGTTTGATCCTG
*Nd-1*	TATCTCAACCCTAGCAGAAA	TAACGCGAATGGGCCGGCTG
*Ifnβ*	CGTGGGAGATGTCCTCAACT	CCTGAAGATCTCTGCTCGGAC
*Cxcl1*	GACCATGGCTGGGATTCACC	GACTTCGGTTTGGGTGCAGT
*Ccl5*	CACCATATGGCTCGGACACC	TCTGGGTTGGCACACACTTG
*Cxcl10*	CGATGACGGGCCAGTGAGAATG	TCAACACGTGGGCAGGATAGGCT
*Gadph*	CTGAGGGAACTGCTGGATAGAG	CGAGGAAACTGGAGCTGCTGAT
*β-actin*	TTCCAGCCTTCCTTCTTG	GGAGCCAGAGCAGTAATC

## Abbreviations

ASC: adaptor apoptosis-associated speck-like protein containing a CARD
AD: Alzheimer's disease
APP: amyloid precursor protein
BCA: bicinchoninic acid assay
cGAS: cyclic GMP-AMP synthase
2'3'-cGAMP: cyclic GMP-AMP
STING: cyclic GMP-AMP receptor stimulator of interferon genes
CsA: cyclosporine A
DRP1: GTPase dynamin-related protein 1
dsDNA: double-stranded DNA
H&E: hematoxylin and eosin
IL-1β: interleukin-1 beta
IRF3: interferon regulatory factor 3
mPTP: mitochondrial permeability transition pore
mtDNA: mitochondrial DNA
Cytb: Mitochondrial cytochrome b
D-loop: Mitochondrial displacement loop
ND1: Monoclonal Antibody to NADH Dehydrogenase 1
NLRP3: NOD, LRR and pyrin domain-containing 3
NF-κB: nuclear factor-κB
OPA1: optic atrophy1
PND: Perioperative neurocognitive disorders
POCD: postoperative cognitive dysfunction
POD: postoperative delirium
PBS: phosphate-buffered saline
p-Tau^T231^: phosphorylated Tau at threonine-231
TBK1: TANK-binding kinase 1
VDAC: voltage-dependent anion channels
VBIT-4: VDAC oligomerization inhibitor
