# Supplementary material for: mtDNA-cGAS-STING axis-dependent NLRP3 inflammasome activation contributes to postoperative cognitive dysfunction induced by sevoflurane in mice
Source: Int J Biol Sci. 2024 Mar 3;20(5):1927–46. doi: 10.7150/ijbs.91543 (PMC10929193; doi:10.7150/ijbs.91543)
Supplement: Supplementary file 1 — Supplementary figures. [file ijbsv20p1927s1.pdf]

Supplementary Figure and Figure legend

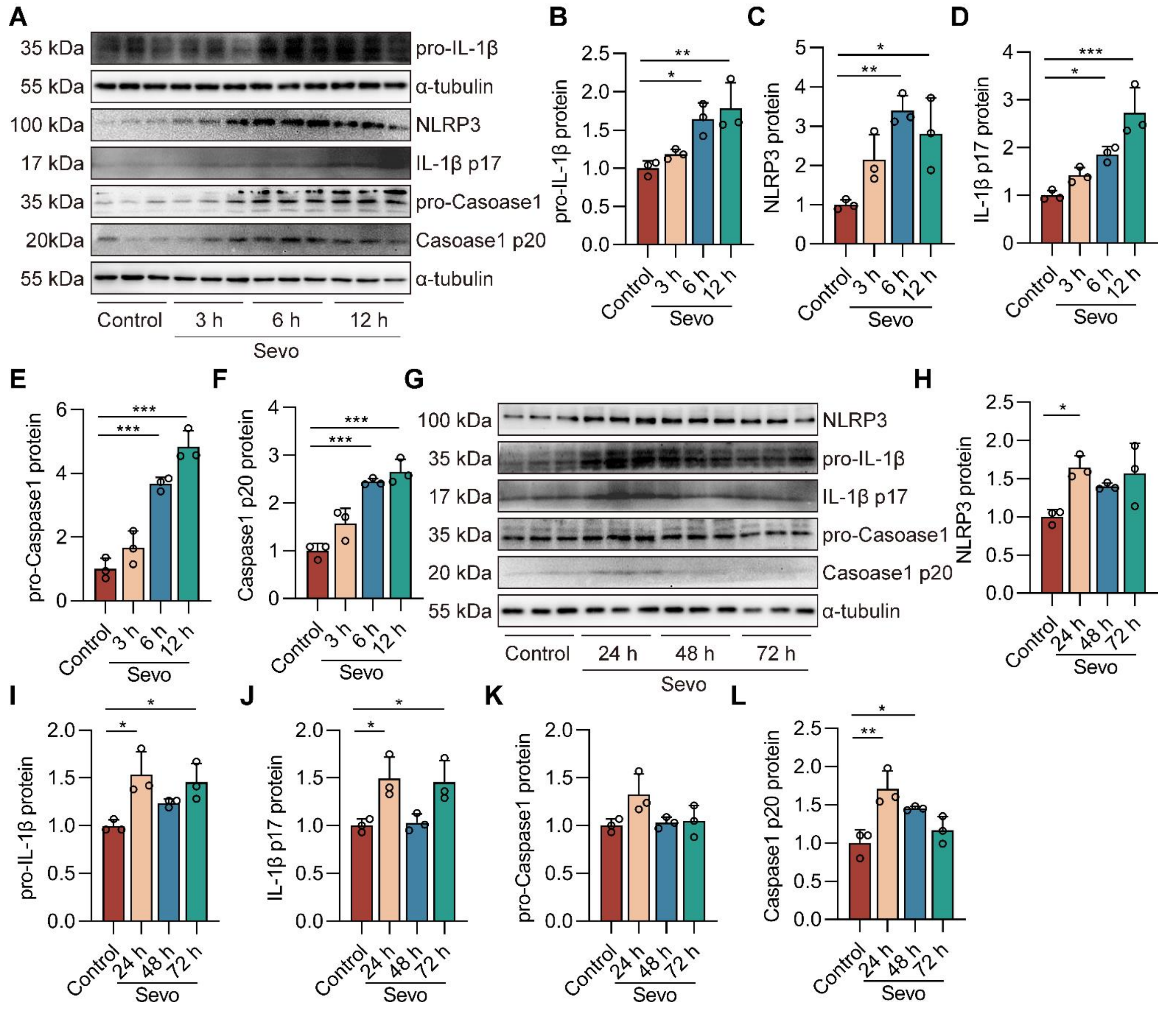

Figure S1. Sevoflurane led to the activation of the NLRP3 inflammasome in microglia at different times. **A-F** BV2 cells were treated with 1 mM Sevoflurane for 3, 6, and 12 h, and the pro-IL-1β, NLRP3, IL-1β p17, pro-Caspase1, and Caspase1 p20 levels were measured by Western Blot (n=3). **G-L** BV2 cells were treated with 1 mM Sevoflurane for 24, 48, and 72 h. The protein expression of NLRP3, pro-IL-1β, IL-1β p17, pro-Caspase1, and Caspase1 p20 were detected by western blot (n=3). Data are expressed as the Mean ± SD. Differences among multiple groups were performed using ANOVA. $^*P < 0.05$, $^{**}P < 0.01$, and $^{***}P < 0.001$.

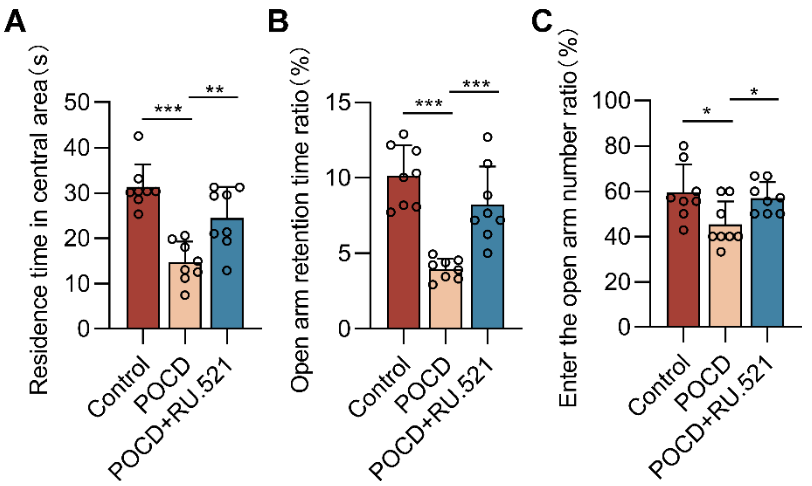

Figure S2. Inhibition of cGAS reduces cognitive impairment in POCD mice. A The Open Field Test was conducted to assess the residence time in the central area (n=8). **B-C** The Elevated Plus Maze was conducted to assess the ratio of time spent in the open arm and the ratio of times entering the open arm (n=8). Data are expressed as the Mean ± SD. Differences among multiple groups were performed using ANOVA. $*P < 0.05$, $**P < 0.01$, and $***P < 0.001$.

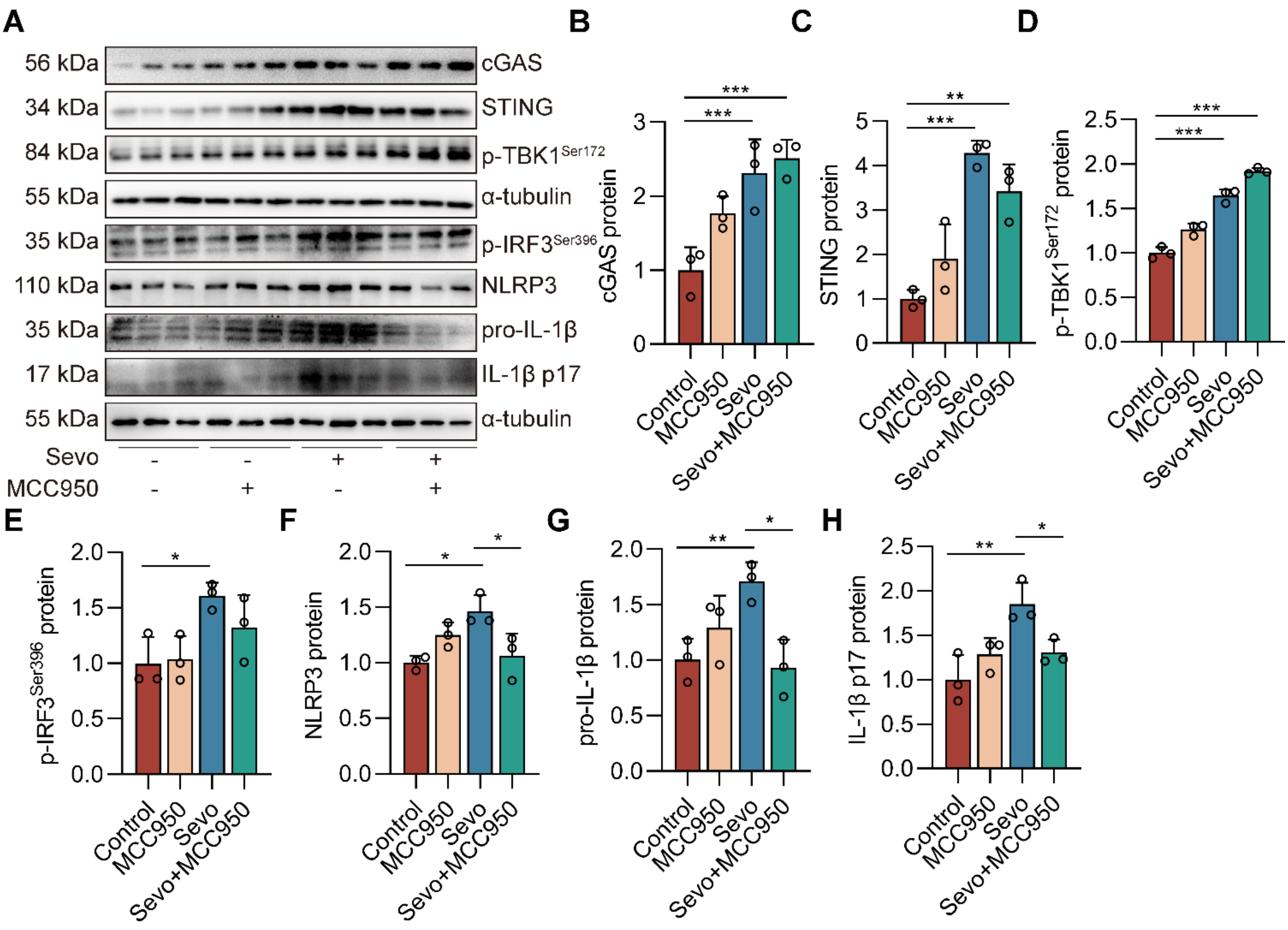

Figure S3. Inhibition of NLRP3 inflammasome has no effect on sevoflurane-induced cGAS-STING pathway activation in microglia. A-H BV2 cells were treated with 1 mM sevoflurane for 12 h after 10 µM

MCC950 intervention for 30 min. The protein expression of cGAS, STING, p-TBK1^{Ser172}, p-IRF3^{Ser396}, NLRP3, pro-IL-1β, and IL-1β p17 levels were measured by Western Blot (n=3). Data are expressed as the Mean ± SD. Differences among multiple groups were performed using ANOVA. *$P < 0.05$, **$P < 0.01$, and ***$P < 0.001$.

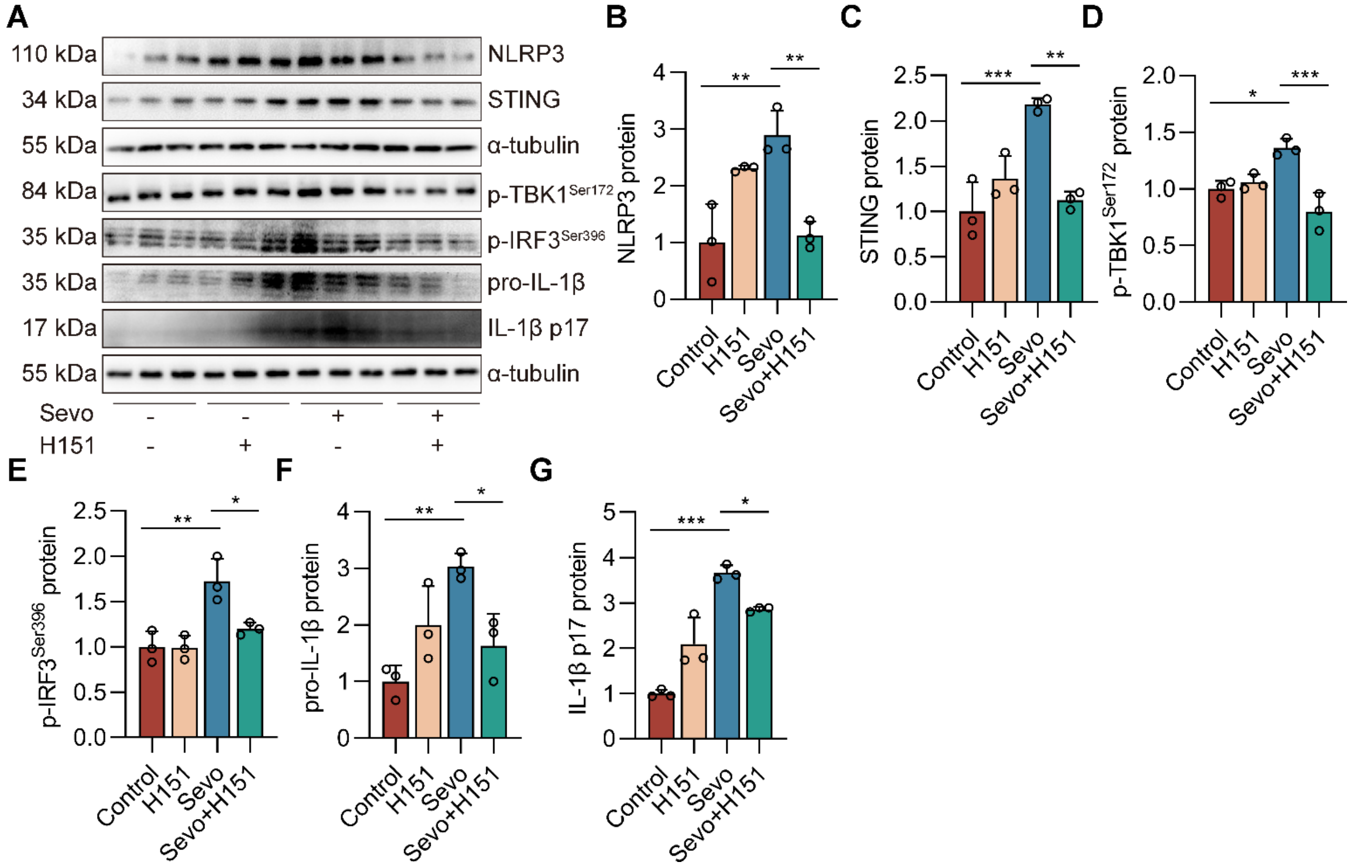

Figure S4. Inhibition of STING suppresses the sevoflurane-induced NLRP3 inflammasome activation in microglia. A-G BV2 cells were treated with 1 mM sevoflurane for 12 h after 5 μM H151 intervention for 30 min. The protein expression of NLRP3, STING, p-TBK1^{Ser172}, p-IRF3^{Ser396}, pro-IL-1β, and IL-1β p17 levels were measured by Western Blot (n=3). Data are expressed as the Mean ± SD. Differences among multiple groups were performed using ANOVA. *$P < 0.05$, **$P < 0.01$, and ***$P < 0.001$.

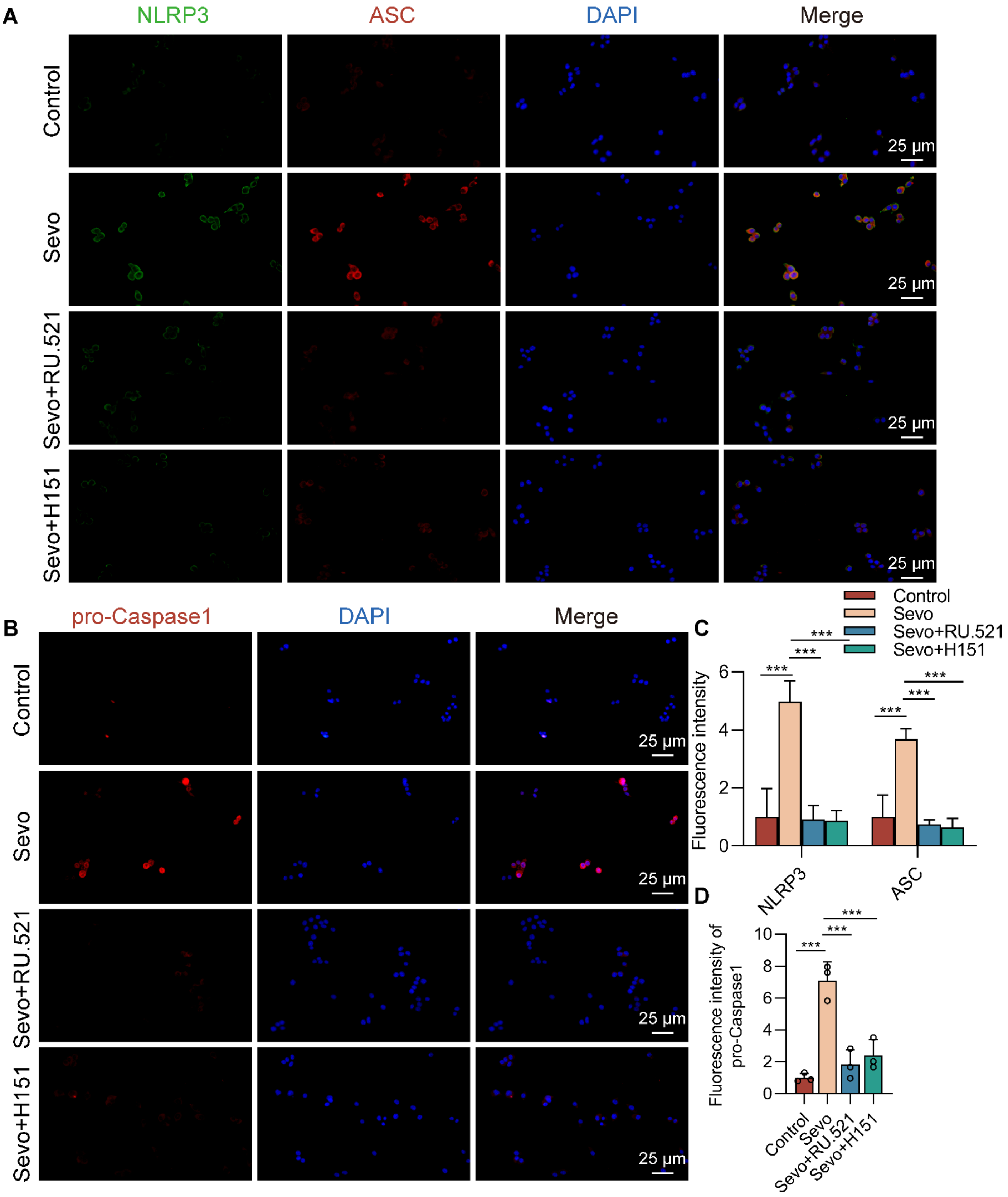

Figure S5. Inhibition of cGAS-STING pathways suppresses the sevoflurane-induced NLRP3 inflammasome activation in microglia. A-D BV2 cells were treated with 1 mM sevoflurane for 12 h after 5 μM H151 intervention for 30 min. The fluorescence intensity of NLRP3, ASC, and pro-Caspase1 were measured by immunofluorescence (*n*=3, bar=25μm). Data are expressed as the Mean ± SD. Differences among multiple groups were performed using ANOVA. ***$P < 0.001$.

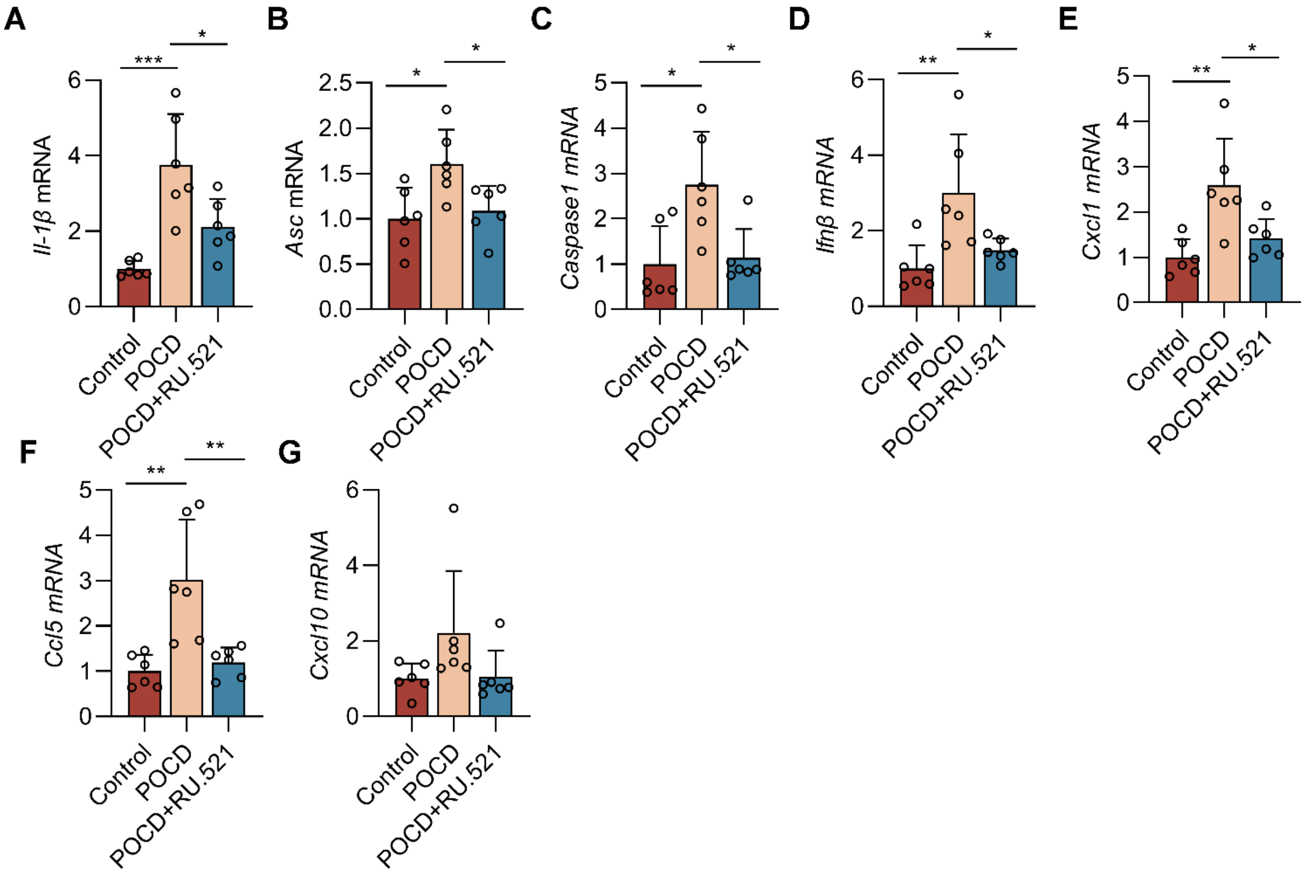

Figure S6. RU.521 inhibits the activation of cGAS-STING pathway downstream genes in POCD mice. **A-G** *Il-1β*, *ASC*, *Caspase1*, *Ifnβ*, *Cxcl10*, *Ccl5,* and *Cxcl10* mRNA expression in the hippocampus were detected by real-time PCR (n=6). Data are expressed as the Mean ± SD. Differences among multiple groups were performed using ANOVA. *$P < 0.05$, **$P < 0.01$, and ***$P < 0.001$.
